# Understanding the Iron-Cobalt Synergies in ZSM-5: Enhanced Peroxymonosulfate Activation and Organic Pollutant Degradation

**DOI:** 10.1021/acsomega.2c01031

**Published:** 2022-05-17

**Authors:** Yaqian Yan, Xinyi Zhang, Jiahao Wei, Miao Chen, Jingtao Bi, Ying Bao

**Affiliations:** † School of Chemical Engineering and Technology, Tianjin University, Tianjin 300072, PR China; ‡ The Co-Innovation Center of Chemistry and Chemical Engineering of Tianjin, Tianjin 300072, PR China; § School of Chemical Engineering and Technology, Hebei University of Technology, No. 8, Guangrong Road, Hongqiao District, Tianjin 300130, PR China

## Abstract

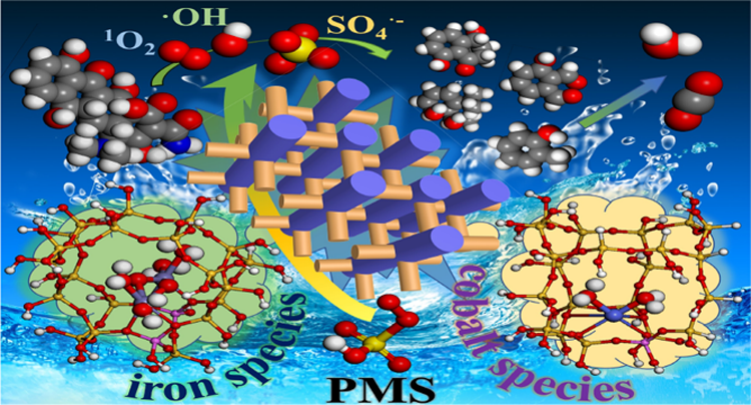

Iron- and cobalt-based heterogeneous catalysts are widely applied for activating peroxymonosulfate (PMS) to degrade organic pollutants. However, few studies have unveiled the clear synergistic mechanism of iron and cobalt in ZSM-5. In this paper, the synergistic mechanism of enhanced PMS activation was revealed by constructing iron and cobalt bimetal modified ZSM-5 zeolite catalysts (FeCo-ZSM-5). The tetracycline hydrochloride (TCH) degradation experiments showed that the catalytic activity of FeCo-ZSM-5-2:3 was much higher than those of Fe-ZSM-5 and Co-ZSM-5. In addition, the influences of catalyst dosage, PMS concentration, reaction temperature, initial pH, and coexisting ions on TCH removal were systematically investigated in this paper. Density functional theory calculations indicated that Co was the main active site for PMS adsorption, and Fe increased the area of Co’s positive potential mapped to the electron cloud. The Fe–Co bimetallic doping increased the area of positive potential mapped to the electron cloud and benefited the adsorption of PMS on the catalyst surface, which revealed the synergistic mechanism of bimetals. Electron paramagnetic resonance spectra and quenching experiments showed that sulfate radicals, singlet oxygen, and hydroxyl radicals were involved in the degradation of TCH. Furthermore, liquid chromatography–mass spectrometry was conducted to propose possible degradation pathways. This work provides certain guiding significance in understanding the synergistic effect of heterogeneous catalysts for tetracycline wastewater treatment.

## 1 Introduction

With economic development, there has been an increase in the production and use of pharmaceuticals and personal care products (PPCPs).^1^ As one of the representative antibiotics, tetracycline antibiotics have been widely applied in animal growth promotion and bacterial infection treatment.^2^ However, such antibiotics are difficult to be completely utilized in humans or animals and will be discharged into the environment.^3^ Traditional physical adsorption,^4^ chemical precipitation,^5^ and biological treatment technologies^6^ cannot effectively destroy tetracycline, and the residuals still pose potential threats to human health and the environment. Therefore, it is of significant necessity to remove hazardous PPCPs from wastewater.

Advanced oxidation processes (AOPs) are efficient ways for oxidizing and degrading PPCPs.^7,8^ The practical application of Fenton system based on •OH is always limited by strict pH requirements.^9^ AOPs based on peroxymonosulfate (PMS) can activate and generate sulfate radicals (SO_4_^•–^), which have the advantages of a wide range of pH, a high oxidation potential, a long half-life time, and biodegradability.^10,11^ Therefore, PMS has been extensively studied in real industrial wastewater, such as landfill leachate,^12^ petrochemical wastewater,^13^ pharmaceutical wastewater,^14^ and pulp and paper wastewater.^15^ Besides, PMS can be activated by many methods, such as thermal activation and UV activation; but they incur high cost and consume more energy. Transition metals (Fe, Co, Cu, and Mn) can activate PMS at room temperature and pressure without additional energy. There are many reports that activated PMS can effectively degrade tetracycline antibiotics.^16,17^ Furthermore, studies have shown that the good dispersion of transition metals on supports can improve the efficiency of catalyst activation of PMS.^18^ Therefore, functional transition metal atoms have been introduced into the zeolite framework to increase the dispersion of catalytically active sites.^19,20^

Aluminosilicate zeolites are regarded as one of the best supports due to its high surface areas and excellent stability.^21^ In the past few years, ZSM-5 zeolite has been intensively employed as the support for constructing highly efficient catalysts because of its high activity, uniform pore structure, excellent hydrothermal stability, and efficient cation exchange ability.^22−25^ With the advantage of low cost and environment friendliness, iron species is an appealing candidate to be doped into the ZSM-5 zeolite.^26,27^ Nonetheless, the poor stability and low transformation efficiency of Fe^3+^/Fe^2+^ inhibit the catalytic activity for PMS activation.^28^ To further improve the activity of iron-based catalysts, it is feasible to add another metal. Co is a good candidate, whose catalytic activity is higher than single-metal catalysts.^20^ For example, it has been reported that iron-cobalt layered double hydroxide can be synthesized to activate PMS to degrade tetracycline. The synergistic effect of Fe and Co made the catalytic reaction more efficient.^29^ However, there are few reports on the application of Fe–Co bimetal-modified ZSM-5 zeolites for wastewater treatment. Furthermore, the deeper synergetic mechanism is still unclear and needs to be revealed.

The application of bimetal cocatalysts has made great progress in dry reforming of methane,^30^ pyrolysis of plastic,^31^ naphtha reforming,^32^ and gas purification.^33^ Different preparation methods of bimetallic catalysts have great influence on the properties and catalytic performance of catalysts.^34^ At present, bimetallic catalysts are mainly prepared by impregnation,^35^ coprecipitation,^36^ plasma treatment,^37^ sol–gel,^38^ and microemulsion methods.^39^ Among them, plasma treatment shows excellent catalytic performance but requires expensive operation equipment. Other preparation methods have been widely used on a laboratory scale and have the potential for future industrial applications.^30^

In this paper, Fe–Co bimetal-modified FeCo-ZSM-5 catalysts were efficiently prepared by the equal volume impregnation method^40^ and characterized. Tetracycline hydrochloride (TCH) is a typical representative of tetracycline antibiotics. The catalytic performances of catalysts were investigated by activating PMS for TCH degradation. The concentration of TCH was determined by a UV–vis spectrophotometer.^41^ The influence of various experimental conditions on TCH removal were systematically studied. Besides, the degradation mechanism of TCH by FeCo-ZSM-5 was explored, and the probable degradation pathways of TCH were put forward. At present, there are few reports on the application of metal-modified ZSM-5 catalysts for organic degradation. FeCo-ZSM-5 catalysts have the advantages of iron and cobalt. Meanwhile, due to the high specific surface area and uniform pore structure of ZSM-5, metal ions are highly dispersed on the surface of ZSM-5, which is conducive to the activation of PMS. Furthermore, the synergistic effect of Fe–Co bimetal doping in ZSM-5 was revealed by density functional theory (DFT) calculations and experiments. This study indicated that FeCo-ZSM-5 materials possess high potential in the PMS activation treatment of tetracycline antibiotic wastewater.

## 2 Materials and Experimental Section

### 2.1 Materials and Chemicals

ZSM-5 was supplied by Tianjin Seans Biochemical Technology Co., Ltd. Ferric nitrate nonahydrate (Fe(NO_3_)_3_·9H_2_O, 98 wt %) and methanol (MeOH, ≥ 99.5 wt %) were provided by Tianjin Komiou Chemical Reagent Co., Ltd. Cobalt nitrate hexahydrate [Co(NO_3_)_2_·6H_2_O, 98 wt %] was obtained from Shanghai Saen Chemical Technology Co., Ltd. Sulfuric acid (H_2_SO_4_, ≥99.7 wt %) and sodium hydroxide (NaOH, 98 wt %) were supplied by Shanghai Myrell Chemical Technology Co., Ltd. TCH (≥ 98 wt %) was obtained from Shanghai Macleans Biochemical Technology Co., Ltd. Tianjin Guangfu Fine Chemicals Co., Ltd. provided sodium bicarbonate (NaHCO_3_, ≥99.5 wt %) and sodium dihydrogen phosphate (KH_2_PO_4_, ≥99 wt %). PMS (2KHSO_5_·KHSO_4_·K_2_SO_4_), tert-butyl alcohol (TBA, ≥ 99 wt %), sodium nitrate (NaNO_3_, ≥99 wt %), and l-histidine (l-his, ≥ 99 wt %) were supplied by Tianjin Kermel Fine Chemicals Co., Ltd. All chemicals were used as purchased without further purification.

### 2.3 Analytical Methods

Powder X-ray diffraction (PXRD) was carried out on a D/MAX 2500 diffractometer (Rigaku, Japan) using Cu Kα radiation (1.5405 Å). The samples were scanned from 2 to 80° (2θ), and the scanning speed was 8° min^–1^. Fourier transform infrared (FT-IR) spectra were analyzed on the Bruker alpha II (Germany) spectrometer. The instrument resolution was 4 cm^–1^, and the test range was 380–2500 cm^–1^. The nitrogen adsorption–desorption isotherms were obtained via Brunauer–Emmett–Teller (BET) method using an SSA-7000 physical adsorption analyzer (China). X-ray photoelectron spectroscopy (XPS) was performed using a Thermo-Fisher ESCALAB-250 Xi spectrometer (UK). The binding energies were calibrated using the C 1s peak at 284.6 eV. The morphology of the catalysts was measured by field emission scanning electron microscopy (FEI Quanta 650 FEG, Japan), and the elemental distribution was analyzed by using energy-dispersive spectroscopy (EDS, Japan). Electron paramagnetic resonance (EPR) technique was detected by a spectrometer (Bruker, EMXPLUS, Germany) using 5,5-dimethyl-1-pyrroline-N-oxide (DMPO) and 2,2,6,6-tetramethyl-4-piperidone (TEMP) as the spin-trapping agents. The residual concentration of TCH was measured via a UV–vis spectrophotometer (UV-3600, Japan). The concentrations of iron and cobalt leaching were quantified by inductively coupled plasma–mass spectrometry (ICP–MS, Agilent 7700s, USA). The total organic carbon (TOC) was measured by a TOC analyzer (Shimadzu, TOC-L, Japan). The degradation pathway of TCH was analyzed by using liquid chromatography–mass spectrometry (LC–MS, Agilent 1290 UPLC/6550 Q-TOF, USA) technology. The mobile phase was 0.1% formic acid aqueous solution/methanol (19:1, v/v) with a flow rate of 0.3 mL/min and column temperature of 25 °C. The injection volume of samples was 2 μL. TCH and the intermediates were estimated in the positive ion mode using electrospray ionization under the following conditions: sheath gas temp, 350 °C; sheath gas flow, 12 L/min; drying gas flow rate, 15 L/min.

### 2.2 Preparation of FeCo-ZSM-5

The methods of metal-modified catalysts include equal volume impregnation^40^ and excessive impregnation.^42^ FeCo-ZSM-5 catalysts were prepared by the equal volume impregnation method. The ZSM-5 zeolite used in the experiment was purchased directly without any treatment. First, Co(NO_3_)_2_·6H_2_O and Fe(NO_3_)_3_·9H_2_O (Table 1) were dissolved in 5.25 mL of deionized water in proportion. Then, the solution was slowly added to 5.00 g of ZSM-5 zeolite and stirred evenly. Then, the samples were placed in an evaporation dish at 25 °C for 24 h and dried at 60 °C for 10 h in a vacuum oven. Finally, the products were calcined at 550 °C for 4 h, and the heating rate was 5 °C/min to obtain FeCo-ZSM-5-*x* (The total metal ion content is 10 wt %. *x* represents the mass ratio of Fe to Co; Fe/Co = 1:0; 2:1; 3:2; 1:1; 2:3; 1:2; and 0:1; the real contents of metal doped are shown in Table S1).

**Table 1:** Mass of Metal Added to FeCo-ZSM-5-*x*

*m* (Fe)/*m* (Co)	1:0	2:1	3:2	1:1	2:3	1:2	0:1
*m* (Fe(NO_3_)_3_·9H_2_O) (g)	3.61	2.40	2.16	1.80	1.44	1.20	0
*m* (Co(NO_3_)_2_·9H_2_O) (g)	0	0.82	0.99	1.23	1.48	1.64	2.47

### 2.4 Experiments and Theoretical Calculation Method

The TCH removal experiments were divided into two steps. First, 0.05 g of catalysts were added into 0.10 L of 20.00 mg/L TCH solution with continuous magnetic stirring for 60 min at a constant temperature of 25 °C. The initial pH was adjusted with 0.1 M H_2_SO_4_ solution or 0.1 M NaOH solution. Then, 0.06 g of PMS was mixed into the above solution and stirred for 60 min. 1 mL of the sample was withdrawn at certain time intervals and mixed with 1 mL of methanol immediately to quench the reaction. The concentration of TCH was conducted by the spectrophotometric method.^43^ To evaluate the reusability and stability of FeCo-ZSM-5-2:3, the used FeCo-ZSM-5-2:3 was collected by filtration, washed thoroughly with deionized water, and dried at 60 °C for 10 h. The recycled particles were reused according to the degradation experiment mentioned above and repeated several times. The pseudo-first-order kinetics model was fitted to describe the catalytic degradation process
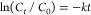
1where *C*_0_ (mg/L) represents the concentration of the initial TCH (after adsorption for 60 min) and *C*_*t*_ (mg/L) is the concentration of TCH at the given reaction times (after adsorption for 60 min). The curve of ln(*C*_*t*_/*C*_0_) versus time was plotted to calculate the slope value, namely, the degradation rate constant *k*.

All experiments were repeated three times, and the error bars are shown in the figures.

All simulations were performed on Materials Studio 5.5 (Accelrys Software Inc., US). The first-principles calculations were carried out based on spin-polarized DFT using DMol3. It should be noted that the ilmenite ZSM-5 crystal belongs to the *Pnma* space group with the lattice parameters of *a* = 1.988 nm, *b* = 2.011 nm, *c* = 1.337 nm, and α = β = γ = 90°. The initial crystal structure data of ZSM-5 were obtained from the website of International Zeolite Association. CASTEP was used for structural optimization, and two optimized 10-member ring structures were selected to construct the cluster model. The initial active species form of Co was Co atom coordinated with four lattice oxygen and two water molecules,^24^ and the initial active species form of Fe was [(H_2_O)_2_–Fe(III)–(μO)_2_–Fe(III)–(H_2_O)_2_]^2+^.^44^

The exchange–correlation functional under the generalized gradient approximation with norm-conserving pseudopotentials and Perdew–Burke–Ernzerhof functional was adopted to describe the electron–electron interaction. The DFT + *U* correction was used in all calculations. A force tolerance of 0.002 Ha Å^–1^, energy tolerance of 1.0 × 10^–5^ Ha per atom, and maximum displacement of 0.005 Å were considered. It should be noted that the system containing Fe and Co needs to consider the influence of electron spin. In addition, in order to improve the calculation accuracy of thermodynamic properties of transition metals, TS method for DFT-D correction is adopted. In the self-consistent field (SCF) calculation, SCF tolerance is set to 1.0 × 10^–6^, multipolar expansion is hexadecapole, and DIIS and smearing are used to speed up SCF convergence. Then, PMS is absorbed on the active species. The adsorption energy was evaluated by the following equation
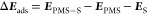
2where the ***E***_PMS-S_, ***E***_PMS_, and ***E***_S_ represent the total energy of PMS adsorbed on the substrate, PMS molecule, and substrate, respectively. According to this equation, a negative Δ***E***_ads_ value indicates that the adsorption process is energetically favorable.

## 3 Results and Discussion

### 3.1 Characterization of Synthesized Catalysts

**Figure 1:**
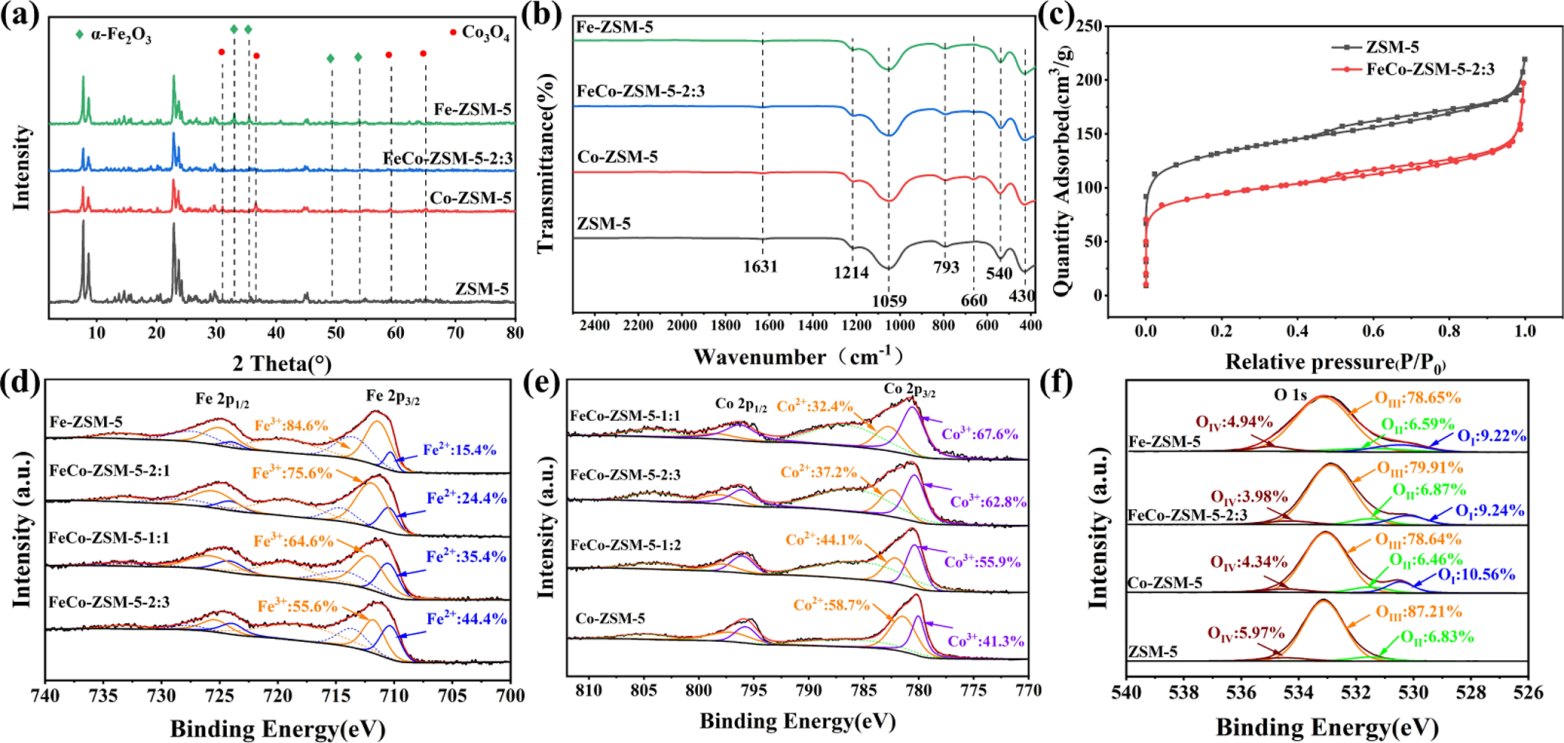
(a) PXRD patterns of FeCo-ZSM-5-*x*. (b) FT-IR spectra of FeCo-ZSM-5-*x*. (c) N_2_ adsorption–desorption isotherms of the catalysts. (d) Fe 2p, (e) Co 2p, and (f) O 1s XPS spectra.

The PXRD patterns of the synthesized samples are shown in. Figures 1a and S1. The characteristic diffraction peaks of ZSM-5 were observed at 7.9, 8.9, 23.1, 23.4, and 24.1°, indicating that the peak positions of ZSM-5 were invariable after incorporation of Fe and Co, and the framework of zeolites was still maintained without any structural change. However, the crystallinity of ZSM-5 was decreased with the addition of Fe and Co, which may be due to the small lattice distortions caused by the intercalation of the metal ions into the framework.^45^ No diffraction peaks characteristic of Fe and Co phases could be observed from the PXRD of FeCo-ZSM-5-2:3, suggesting the high dispersion of these metals in the catalysts. When the percentage of Fe exceeded 50%, the characteristic peaks of α-Fe_2_O_3_ were observed at 33.1, 35.6, 49.4, and 54.0°.^46,47^ Similarly, the characteristic peaks of Co_3_O_4_ were also observed at 31.2, 36.8, 59.3, and 65.2° with the proportion of Co increased to 66.7%.^48,49^ The phenomena above indicated that Fe_2_O_3_ and Co_3_O_4_ crystals were generated under the condition of high metal-ion loading.

The FT-IR spectra of FeCo-ZSM-5-*x* are shown in Figures 1b and S2. The bands at 430, 793, and 1059 cm^–1^ could be assigned to the T–O (T is Si or Al) bending vibrations of internal tetrahedral, the symmetric stretching vibration of the T–O bond, and the asymmetrical stretching vibration of the T–O–T bond, respectively.^49^ The peak at 550 cm^–1^ was a unique vibration band of ZSM-5 corresponding to the double five-membered ring vibration, and the band at about 1220 cm^–1^ was attributed to the asymmetric stretching of external linkages of the tetrahedrons.^50^ After deposition of Fe and Co, the skeleton structure of ZSM-5 was still preserved, which was consistent with the PXRD data (Figures 1a and S1). Furthermore, the peak at 660 cm^–1^ was attributed to the Co–O stretching vibration of Co_3_O_4_ in Co-ZSM-5 and FeCo-ZSM-5-1:2.^51^ However, there was no obvious peak of Fe_2_O_3_ at 540 cm^–1^ owing to the overlap with ZSM-5 peaks.

The BET and BJH methods were used to analyze the surface areas and pore size distribution of FeCo-ZSM-5-2:3 (Figures 1c and S3). The N_2_ physical adsorption of the catalysts can be categorized as type-I isothermal adsorption with the H4-type hysteresis ring, indicating the microporous nature of the catalysts.^46^ With the addition of Fe and Co, the specific surface and the pore volume decreased slightly, which further indicated that metal ions entered into ZSM-5.

XPS is used to analyze the existing forms of Fe, Co, and O elements in FeCo-ZSM-5 catalysts (Figure S4), and the detailed fitting parameters for the XPS spectra are shown in. Table S2. The acquired spectra were calibrated by standard carbon C 1 s (284.6 eV). The characteristic peaks of Fe 2p_3/2_ (712.0 eV) and Fe 2p_1/2_ (726.0 eV) and two corresponding satellite peaks were observed in the Fe 2p XPS spectra (Figure 1d). The binding energy of Fe 2p_3/2_ can be deconvoluted into 711.6 and 710.3 eV, which were assigned to Fe^3+^ and Fe^2+^ species, respectively.^52^Figure 1e describes the XPS spectra of Co 2p. The fitting peaks at 781.0 and 796.0 eV belonged to Co 2p_3/2_ and Co 2p_1/2_. The binding energy of Co 2p_3/2_ can be fitted into two main peaks, which were classified as Co^2+^ and Co^3+^ species, respectively.^53^ It can be seen that as the ratio of the Co element improved, the proportion of Fe^2+^ increased, while the percentage of Co^2+^ decreased. The Fe^2+^/Fe^3+^ ratio was 0.8 and Co^2+^/Co^3+^ ratio was 0.6 in the FeCo-ZSM-5-2:3. This may be caused by two reasons. First, Fe^3+^ and Co^2+^ may produce redox reactions to form mixed oxides during the calcination.^54^ Second, there was charge transfer between metals and the zeolite framework,^55^ and the electrons of Co^2+^ may flow to Fe^3+^ along the zeolite framework.

Figure 1f exhibits the XPS spectra of O 1s, which existed in three forms: hydroxy oxygen (O_IV_) at 534.5 eV, lattice oxygen (O_III_) at 533.1 eV, and adsorbed oxygen (O_II_) at 531.6 eV.^56,57^ After the zeolite was loaded with metal ions, the emergence of binding energy at 530.2 eV can be attributed to the metal lattice oxygen (O_I_). Experimental data indicated that the O_II_ content of FeCo-ZSM-5-2:3 was higher than that of other catalysts,^56^ which was conducive to the activation of PMS, thus improving the removal efficiency.

**Figure 2:**
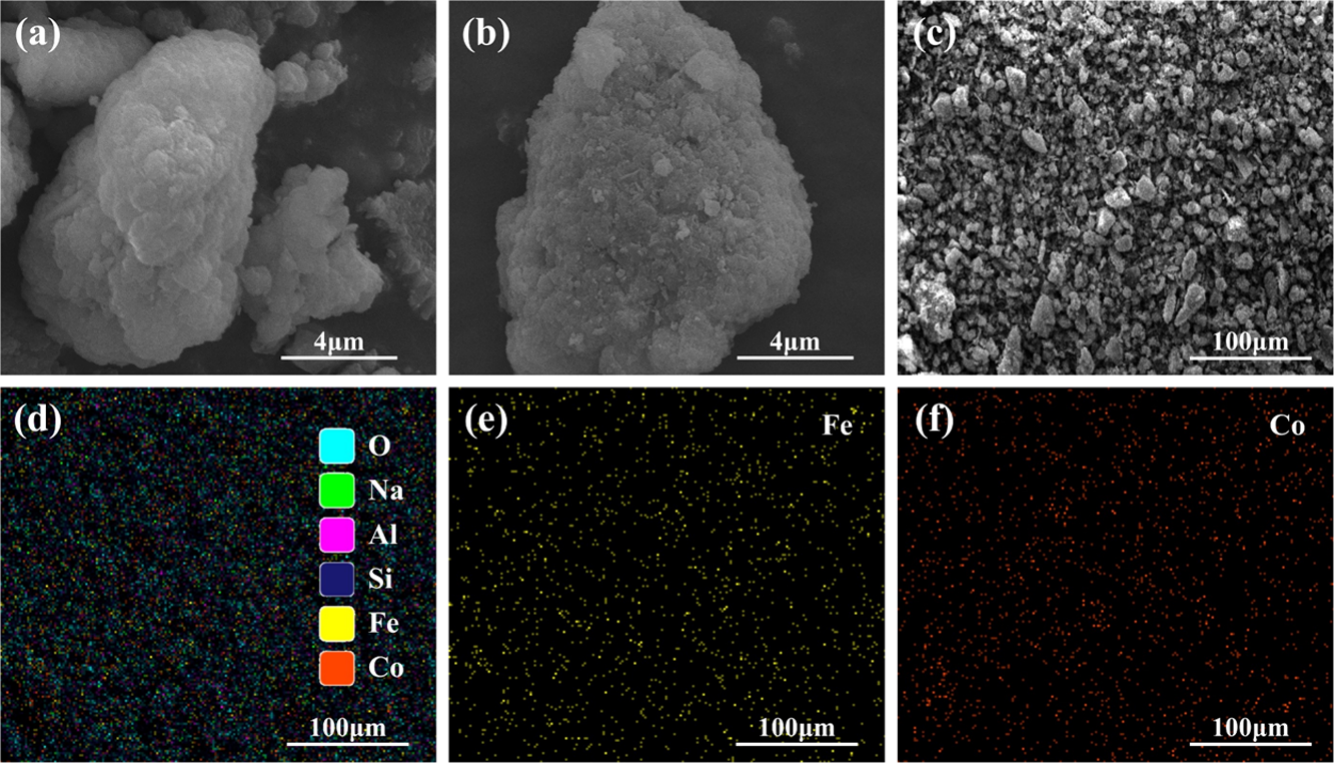
SEM images of different catalysts: (a) ZSM and (b) FeCo-ZSM-5-2:3. (c–f) EDS elemental mapping images of FeCo-ZSM-5-2:3.

The SEM images of FeCo-ZSM-5-*x* are shown in Figures 2 and S5. There was no significant change in the morphology as ZSM-5 was loaded with Fe and Co (Figure 2a,b), which demonstrated the microstructure stability of ZSM-5 before and after loading. The results are consistent with the observation of PXRD (Figures 1a and S1) and FT-IR (Figures 1b and S2). Furthermore, the elemental distribution of FeCo-ZSM-5-*x* was obtained via EDS mapping (Figures 2c–f, S6 and Table S3).

### 3.2 Catalytic Performance

**Figure 3:**
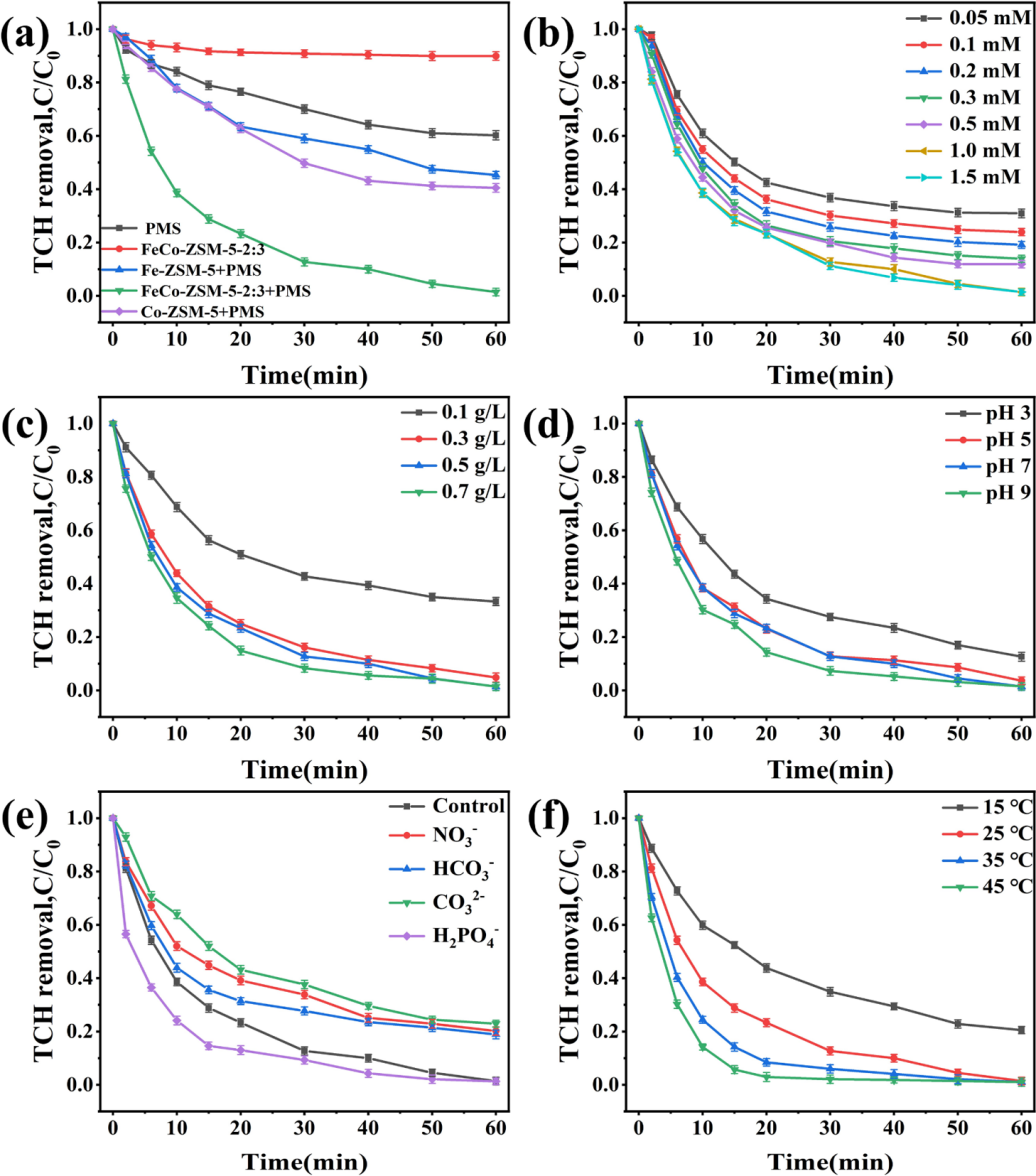
(a) Degradation of TCH in different reaction systems. Effects of (b) concentration of PMS, (c) dosage of catalysts, (d) initial pH, (e) coexisting anions, and (f) temperature on TCH degradation by FeCo-ZSM-5-2:3/PMS system. General conditions: [pollutant] = 20 mg/L for (a–f); [PMS] = 1 mM for (a and c–f); [initial pH] = 7.0 for (a–c and e–f); [catalyst] = 0.5 g/L for (a,b and d–f); [coexisting anions] = 0.2 mol/L for (e); and reaction temperature is 25 °C for (a–e).

According to the literature,^58^ organic pollutants also can be directly oxidized by unactivated PMS due to the inherent high redox potential [*E*^0^ (HSO_5_^–^/HSO_4_^–^) = +1.82 V_NHE_]. As shown in Figures 3a and S7, the addition of PMS alone resulted in 40% removal of TCH in 60 min. The TCH removal rates of Fe-ZSM-5 and Co-ZSM-5 within 60 min were 54.7 and 59.5%, respectively. With the increase of Fe and Co dosage, the catalytic performance of FeCo-ZSM-5-*x* was improved, which reflected the bimetal synergistic effects. FeCo-ZSM-5-2:3 exhibited the optimum catalytic activity (removal rate of TCH reached 98.6% within 60 min). Moreover, it was observed that FeCo-ZSM-5-2:3 had a low adsorption capacity on TCH (only 10%). The degradation rate constants *k* (eq 1) of TCH removal were calculated (Figure S8). FeCo-ZSM-5-2:3 showed the highest *k* value, which may be due to the high dispersion of the metal on FeCo-ZSM-5-2:3, and significantly enhances the catalytic activity.

The effect of PMS dosage on TCH removal^59^ is investigated in the range of 0.05–1.5 mM (Figure 3b). It can be observed that the removal of TCH improved from 69.1 to 98.3% as the concentration of PMS increased from 0.05 mM to 1 mM.^60^ Nevertheless, there was no significant increase in the TCH removal efficiency when the PMS dosage further reached 1.5 mM. This might be because excessive PMS would lead to scavenging of reactive oxygen species (ROS), producing low reactive radicals (eqs 3 and 4).^61,62^ Therefore, the PMS concentration was selected to be 1 mM for subsequent experiments.
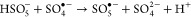
3
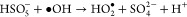
4

Figure 3c shows the effect of catalyst dosage on the removal of TCH. When the catalyst dosage was promoted from 0.1 to 0.3 g/L, the TCH removal efficiency increased from 66.7 to 95.2%, and as the catalyst dosage increased from 0.3 to 0.5 g/L, the TCH removal rate increased from 95.2 to 98.6%. Obviously, more active sites for the activation of PMS could be provided by increasing the catalyst dosage. In addition, the removal rate varied little when the catalyst dosage was increased from 0.5 to 0.7 g/L. Thus, from the perspective of cost, 0.5 g/L was selected as the optimal catalyst dosage in this paper.

The pH value plays a crucial role in catalytic degradation since it influences the production of primary principal radical types.^63,64^ The impact of initial pH on TCH removal could be observed in Figure 3d. The zeta potential at different pH values is shown in Table S4. Effective removal of TCH over a wide pH range (3–9) was observed. However, the degradation rate was limited under acidic conditions. As HSO_5_^–^ was dominant in PMS, which existed in the form of H_2_SO_5_, it is hard to be transformed into ROS.^65^ TCH removal efficiency was higher under alkaline conditions due to the enhanced self-decomposition of PMS and the generation of singlet oxygen (^1^O_2_).^66,67^ Furthermore, the TCH removal efficiency was higher in the presence of FeCo-ZSM-5-2:3 catalysts in the PMS/NaOH (pH 9) system (Figure S9), which indicated that the accession of catalysts could activate PMS more effectively. Therefore, the pH was selected to be 7 for subsequent experiments.

Inorganic anions in water could react with free radicals and affect the oxidation of organics by free radicals. In order to confirm the anti-interference ability of the catalysts in the environment, the TCH degradation experiments were carried out under the coexistence (0.2 mol/L) of different anions (HCO_3_^–^, H_2_PO_4_^–^, NO_3_^–^, and CO_3_^2–^) according to the literature^59^ (Figure 3e). First, H_2_PO_4_^–^ promoted the degradation of TCH as the coexisting phosphate could lower the O–O bond dissociation energy in the PMS molecule, which was more conducive to the generation of ROS to degrade organic pollutants.^68^ Contrarily, HCO_3_^–^, CO_3_^2–^, and NO_3_^–^ obviously inhibited the degradation of TCH due to their quenching effects on •OH and SO_4_^•–^, resulting in the decreased efficiency for TCH degradation (eqs 5–10).^69−71^
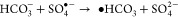
5
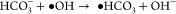
6
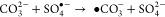
7
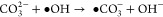
8
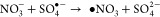
9
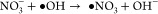
10

Figure 3f presents the influence of temperature on TCH degradation. With the increase of temperature, the degradation efficiency of TCH was greatly improved, which can reach about 95% in 15 min at 45 °C as enough energy was provided by the higher temperature to accelerate the cleavage of PMS molecules to generate free radicals. In addition, the activation energy (*E*_a_) value of FeCo-ZSM-5-2:3 was 56.81 kJ/mol.

**Figure 4:**
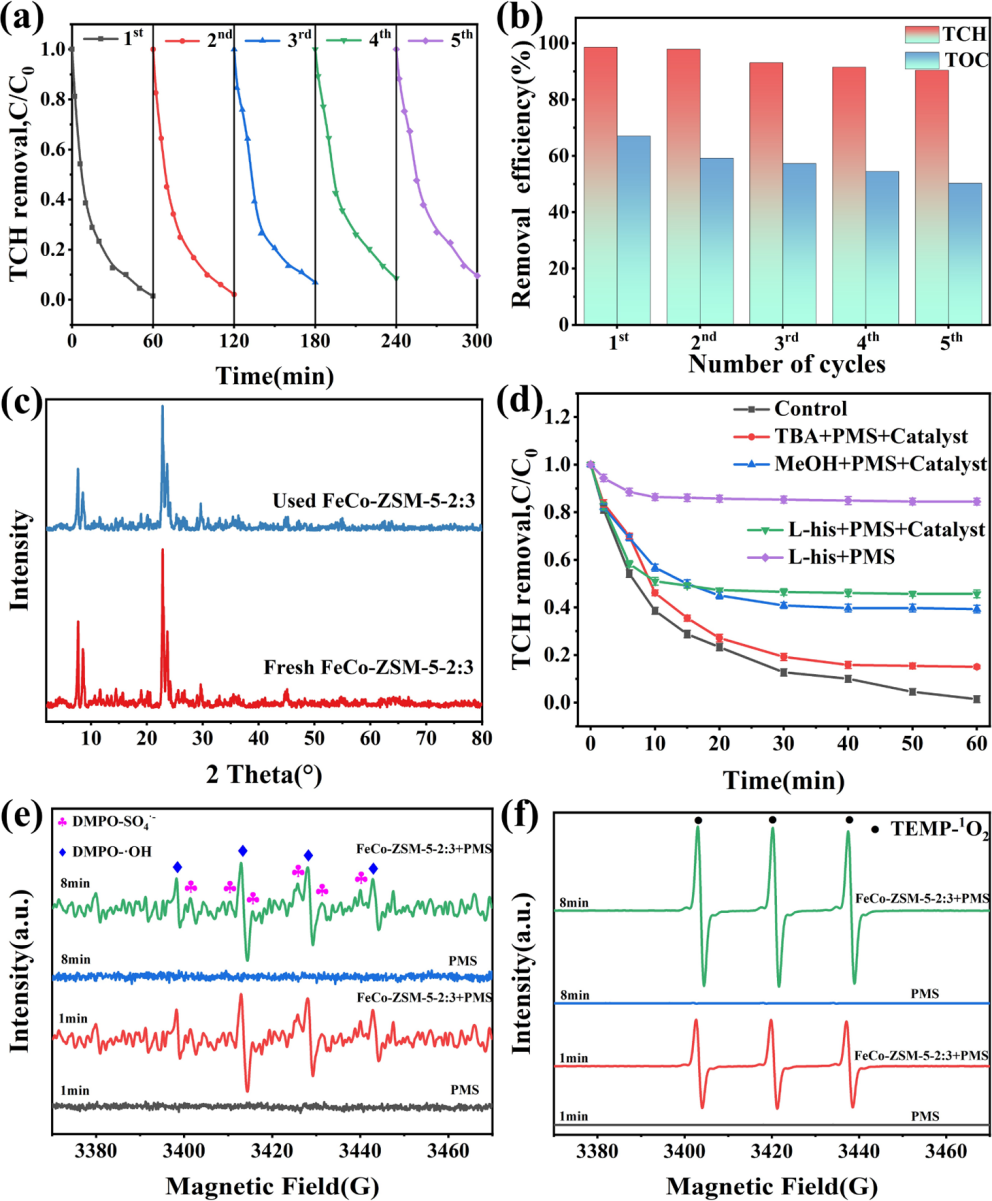
(a) Reusability experiments with recycled FeCo-ZSM-5-2:3. (b) Reusability experiments for TCH removal and TOC removal in different cycles. (c) PXRD of the fresh and used FeCo-ZSM-5-2:3. (d) Quenching tests using different scavengers. (e–f) Identification of reactive species by using EPR spectra. General conditions: [pollutant] = 20 mg/L; [initial pH] = 7.0; [catalyst] = 0.5 g/L; [PMS] = 1 mM; and [temperature] = 25 °C.

As depicted in Figure 4a, the degradation efficiency of TCH still maintained more than 90% after 5 times of use. The results confirmed that the catalyst had well reusability. Furthermore, the TOC removal of FeCo-ZSM-5-2:3 is illustrated in Figure 4b, which was 67.1% at the first use and 50.3% after the fifth use. The leaching of Fe and Co after the reactions is listed in Figure S10. The concentrations of iron ions (<0.21 mg/L) and cobalt ions (<0.86 mg/L) leached from the solution were lower than the allowable emission limits of iron ions (0.3 mg/L) and cobalt ions (1 mg/L) in China’s Environmental Quality Standard of Surface Water (GB3838-2002).^72^

### 3.3 Catalytic Mechanism

The above analysis results show that FeCo-ZSM-5-2:3 has sufficient stability in TCH degradation. Figure 4c shows the PXRD patterns of FeCo-ZSM-5-2:3 after five times of use. The catalysts still had stronger PXRD diffraction peaks, and no new peaks appeared in contrast to the fresh catalysts, which further confirmed the structural stability of FeCo-ZSM-5-2:3 under catalytic oxidation.

The degradation mechanism of TCH and the participation of free radicals were studied by quenching tests. MeOH was selected as a scavenger of •OH and SO_4_^•–^.^73^ TBA was used to capture •OH.^74^L-his was used for quenching ^1^O_2_.^75^ As shown in Figure 4d, the degradation efficiency declined to 85% with the addition of 500 mM TBA, indicating that •OH had limited impacts on the degradation processes. The removal of TCH was reduced to 60.7% with the existence of 500 mM MeOH, which implied that SO_4_^•–^ was more significant for TCH removal. Besides, after 10 mM L-his was added, the degradation efficiency of TCH was reduced to 15% in the sole PMS system, and the degradation efficiency of TCH was reduced to 54.3% in the FeCo-ZSM-5-2:3/PMS system. Obviously, ^1^O_2_ was also produced during PMS activation. Therefore, ^1^O_2_ and SO_4_^•–^ acted as the dominant radicals in the degradation of TCH. In addition, Figure S11 shows the result of the quenching tests under alkaline condition (pH 9), which indicates that there was no significant difference compared with the neutral condition. The produced ROS (•OH, SO_4_^•–^and ^1^O_2_) were considered to react with TCH first, instead of preferentially producing low reactive radicals.

EPR was used to prove the conclusion of the quenching experiments.^59^ As presented in Figure 4e,f, DMPO was used to test •OH and SO_4_^•–^, and TEMP was selected as the trapping agent to test ^1^O_2_. The results revealed that the DMPO-•OH, DMPO-SO_4_^•–^, and TEMP-^1^O_2_ EPR signals verified the generation of •OH, SO_4_^•–^, and ^1^O_2_ in the FeCo-ZSM-5-2:3/PMS system. There were no obvious characteristic peaks in the sole PMS system. With the increase of the reaction time, the signals enhanced, which suggested the accumulation of ROS. Besides, the free radical intensities of •OH, SO_4_^•–^, and ^1^O_2_ in the FeCo-ZSM-5-2:3/PMS system were much higher than those in the Fe-ZSM-5/PMS system and the Co-ZSM-5/PMS system (Figure S12), indicating Fe–Co bimetallic synergistic effects. It can be observed that the coexistence of free radicals and nonfree radical ROS in the FeCo-ZSM-5-2:3/PMS system had wide applicability in the degradation of other pollutants.

**Figure 5:**
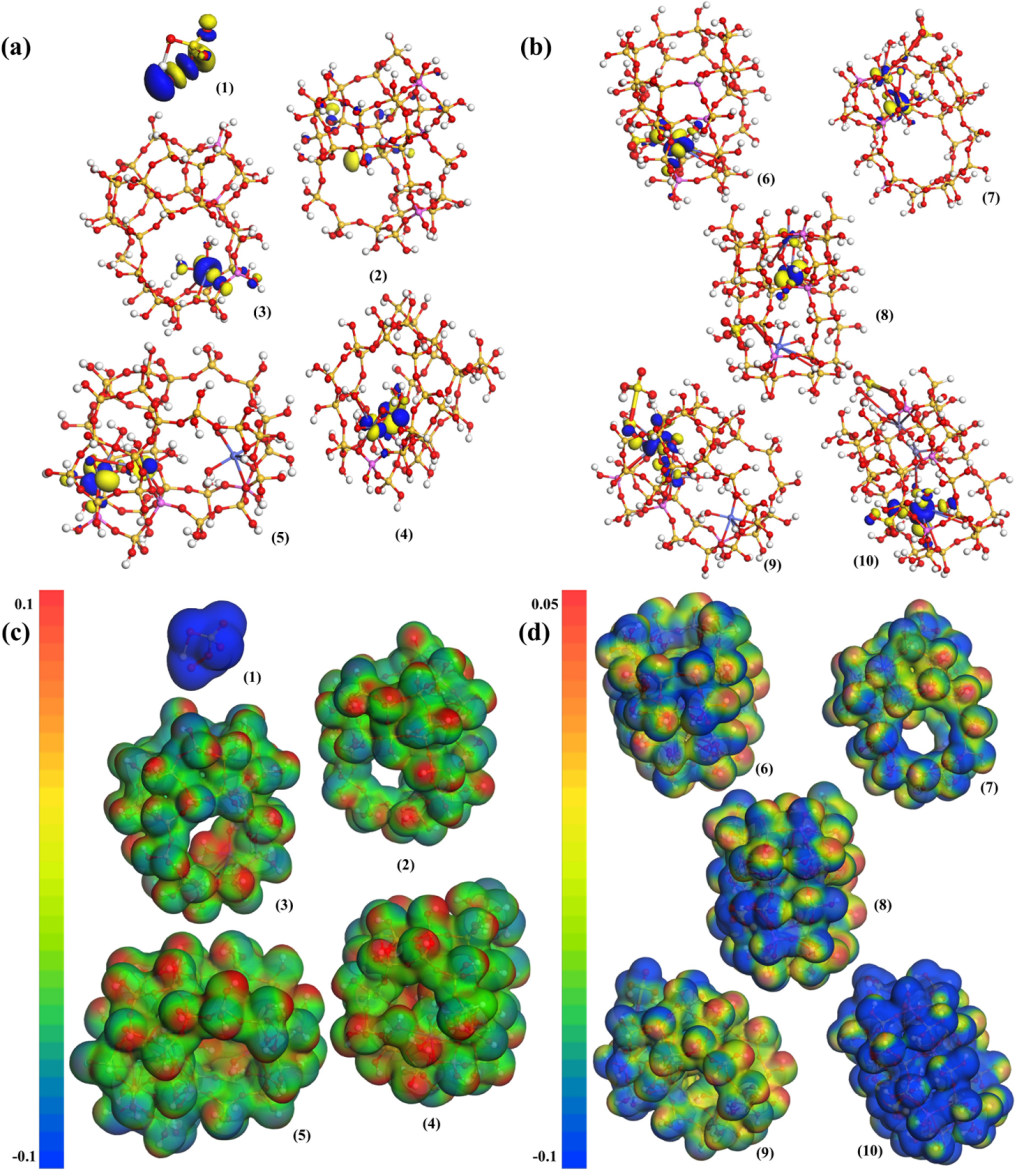
(a) LUMO orbitals for PMS and catalyst cluster models. (b) LUMO orbitals of catalyst-PMS adsorption cluster models. (c) Electrostatic potential diagrams of PMS and catalyst cluster models. (d) Electrostatic potential diagram of catalyst-PMS adsorption cluster models. [(1) PMS, (2) ZSM-5, (3) Co-ZSM-5, (4) Fe-ZSM-5, (5) FeCo-ZSM-5, (6) Co-ZSM-5-PMS, (7) Fe-ZSM-5-PMS, (8) FeCo-ZSM-5-Co-PMS, (9) FeCo-ZSM-5-Fe-PMS, and (10) FeCo-ZSM-5-FeCo-PMS].

To further prove that Fe–Co bimetallic doping had synergistic effects, the cluster model was analyzed by DFT calculations. As can be seen from Figure 5a, the LUMO orbitals of the unmodified ZSM-5 models were symmetrically distributed along the silicon-oxygen bond framework of the zeolites. The LUMO orbitals moved to the vicinity of the active species after the active species of Co and Fe were introduced, which was conducive to the adsorption and activation of the negatively charged PMS. In Figure 5b, as the PMS bound to FeCo-ZSM-5, the LUMO orbital would be transferred to the active species and the PMS binding structure or remained at another active species. The coordinate data of Fe-ZSM-5, Co-ZSM-5, and FeCo-ZSM-5 after optimization are shown in Table S5. The data indicate that the combined products had high reactivity. Meanwhile, other active species without adsorption still had the ability to bind to PMS.

Combined with the electrostatic potential diagram, it could be observed that the PMS had strong electronegativity and could interact with metal active species with positive charges. Figure 5c, eq 3, and eq 4, respectively, show the strong positive charge of the active species of Co and Fe. Meanwhile, it can be seen from Figure 5c and eq 5 that the presence of Co and Fe coexisting ions increased the range of electropositivity, which contributed to enhancing the adsorption capacity of the active species and PMS.^76^ By calculating the total energy of the models before and after the adsorption of PMS, the total energy difference of FeCo-ZSM-5 is lower than that of Co-ZSM-5 (0.86 eV). It could be seen that after the addition of Fe, the area of positive potential mapped by the active species of Co in the electron cloud became larger. Figure 5d shows that the overall potential of the single-metal-modified catalysts decreased significantly and the reaction activity was obviously reduced after PMS adsorption. In FeCo-ZSM-5, Co was the main active site for PMS adsorption. Considering the regulating action of Fe, an appropriate ratio of Fe to Co would achieve higher catalytic effects.

**Figure 6:**
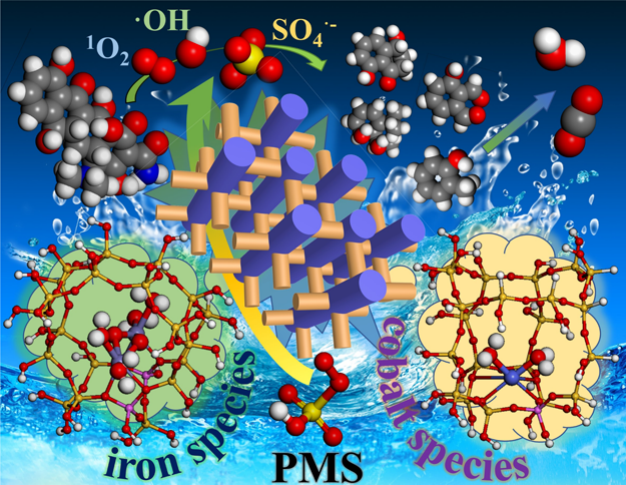
Possible TCH degradation mechanism of the FeCo-ZSM-5-2:3 catalysts.

From both experimental and theoretical studies (Figure 6) the catalytic oxidation mechanism for FeCo-ZSM-5-2:3 to activate PMS to degrade TCH was proposed. It included nonfree radical processes and surface-bound free radical processes. For nonradical processes, the catalysts enhanced the self-decomposition of PMS to produce ^1^O_2_ for TCH degradation as exhibited in eq 11.^77^ On the other hand, for surface-bound radical processes, when FeCo-ZSM-5-2:3 and PMS were added to the aqueous solution, the redox reactions of Co^2+^ → Co^3+^ →Co^2+^ and Fe^3+^ → Fe^2+^ → Fe^3+^ were generated due to the presence of HSO_5_^–^ (eqs 12–15).^78^ During the reactions, SO_4_^•–^ was produced to participate in the reduction of TCH. In addition, the Co^2+^ and Fe^2+^ on the catalyst surface may react with OH^–^/H_2_O to form CoOH^+^ and FeOH^+^, and then the redox reactions of CoOH^+^ → CoOH^2+^ → CoOH^+^ and FeOH^+^ → FeOH^2+^ → FeOH^+^ were generated due to the presence of HSO_5_^–^ to form SO_4_^•–^, respectively (eqs 16–19).^78^ Meanwhile, SO_4_^•–^ could react with OH^–^ to produce •OH (eq 20).^79^
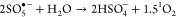
11
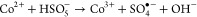
12
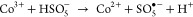
13
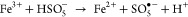
14
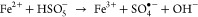
15
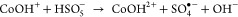
16
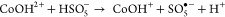
17
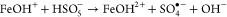
18
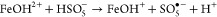
19
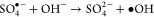
20

### 3.4 Possible Degradation Pathway of TCH

The possible degradation pathway of TCH is shown in Text S1.

## 4 Conclusions

In summary, the bimetal-doped FeCo-ZSM-5 catalysts were prepared by using ZSM-5 zeolite as the carrier for activating PMS to degrade TCH in this paper. Experiments showed that FeCo-ZSM-5-2:3 had the highest catalytic activity, and its degradation rate constants (*k*) of TCH degradation was calculated as 0.0632 min^–1^, which was much higher than those of Fe-ZSM-5 (0.190 min^–1^) and Co-ZSM-5 (0.191 min^–1^). Quenching experiments and EPR suggested that various substances such as ^1^O_2_, SO_4_^•–^, and •OH participated in the degradation processes of TCH. The degradation efficiency and TOC removal efficiency of TCH still maintained more than 90 and 50% after five cycles, respectively, indicating that the catalyst had certain reusability. DFT calculations unveiled the synergistic effects mechanism of Fe and Co, which significantly enhanced the binding ability with PMS by changing the distribution of the positive potential. In addition, LC–MS was used to detect the oxidation intermediates of TCH, and the probable degradation pathways are proposed. FeCo-ZSM-5/PMS system had high catalytic activity; free radicals coexist with nonfree radical ROS. Meanwhile, the catalyst had good reusability. This system has universal applicability for treating other wastewater containing refractory organics.

## Supporting Information Available

PXRD patterns of catalysts; FTIR spectra of catalysts; pore size distribution of catalysts; XPS survey of catalysts; SEM images of catalysts; EDS element composition of FeCo-ZSM-5-*x*; TCH removal by PMS activation on different catalysts; linear fitting of different catalysts on TCH degradation in the presence of PMS; control experiments of PMS/NaOH (pH 9); metal leaching in different cycles; quenching tests using different scavengers under alkaline condition; EPR spectra with DMPO and TEMP; final concentrations of FeCo-ZSM-5-*x* metals loaded by using ICP–MS; detailed fitting parameters for the XPS spectra; EDS element composition of FeCo-ZSM-5-*x*; zeta potential at different pH; coordinate data of Fe-ZSM-5, Co-ZSM-5, and FeCo-ZSM-5; and possible degradation pathway of TCH (PDF)
