# Supplementary material for: Understanding the Iron-Cobalt Synergies in ZSM-5: Enhanced Peroxymonosulfate Activation and Organic Pollutant Degradation
Source: ACS Omega. 2022 May 17;7(21):17811–21. doi: 10.1021/acsomega.2c01031 (PMC9161407; doi:10.1021/acsomega.2c01031)
Supplement: Supplementary file 1 — ao2c01031_si_001.pdf [file ao2c01031_si_001.pdf]

Supplementary Information

Understanding the Iron-Cobalt Synergies in ZSM-5: Enhanced Peroxymonosulfate Activation and Organic Pollutant Degradation

Yaqian Yan [a, b], Xinyi Zhang [a], Jiahao Wei [a, b], Miao Chen [a, b], Jingtao Bi [c], Ying Bao [a, b *]

[a] *School of Chemical Engineering and Technology, Tianjin University, Tianjin 300072, PR China*

[b] *The Co-Innovation Center of Chemistry and Chemical Engineering of Tianjin, Tianjin 300072, PR China*

[c] *School of Chemical Engineering and Technology, Hebei University of Technology, No.8, Guangrong Road, Hongqiao District, Tianjin 300130, PR China*

[*]**Corresponding Author**

Phone: 86-22-27405754; fax: 86-22-27374971

E-mail address: yingbao@tju.edu.cn

Figure S1. PXRD patterns of catalysts. S4

Figure S2. FTIR spectra of catalysts..S5

Figure S3. The pore size distribution of catalysts. S6

Figure S4. XPS survey of catalysts...S7

Figure S5. SEM images of catalysts. S8

Figure S6. EDS element composition of FeCo-ZSM-5-*x*. S9

Figure S7. TCH removal by PMS activation on different catalysts...........................S10

Figure S8. The liner fitted of different catalysts on TCH degradation in the presence of PMS...S11

Figure S9. Control experiments of PMS/NaOH (pH 9)..S12

Figure S10. Metal leaching in different cycles. S13

Figure S11 Quenching tests using different scavengers under alkaline condition.....S14

Figure S12. EPR spectra with DMPO in (a) FeCo-ZSM-5-2:3/PMS and Fe-ZSM-5/PMS systems and (b) FeCo-ZSM-5-2:3/PMS and Co-ZSM-5/PMS systems; EPR spectra with TEMP in (c) FeCo-ZSM-5-2:3/PMS and Fe-ZSM-5/PMS systems and (d) FeCo-ZSM-5-2:3/PMS and Co-ZSM-5/PMS systems. S15

Table S1. The final concentrations of FeCo-ZSM-5-*x* metals loaded by using ICP-MS. S16

Table S2. The detailed fitting parameters for the XPS spectra. S17

Table S3. EDS element composition of FeCo-ZSM-5-*x*. S18

Table S4. Zeta potential at different pH. S19

Table S5. The coordinate data of Fe-ZSM-5, Co-ZSM-5 and FeCo-ZSM-5.............S20

Text S1. Possible degradation pathway of TCH. S27

References..S30

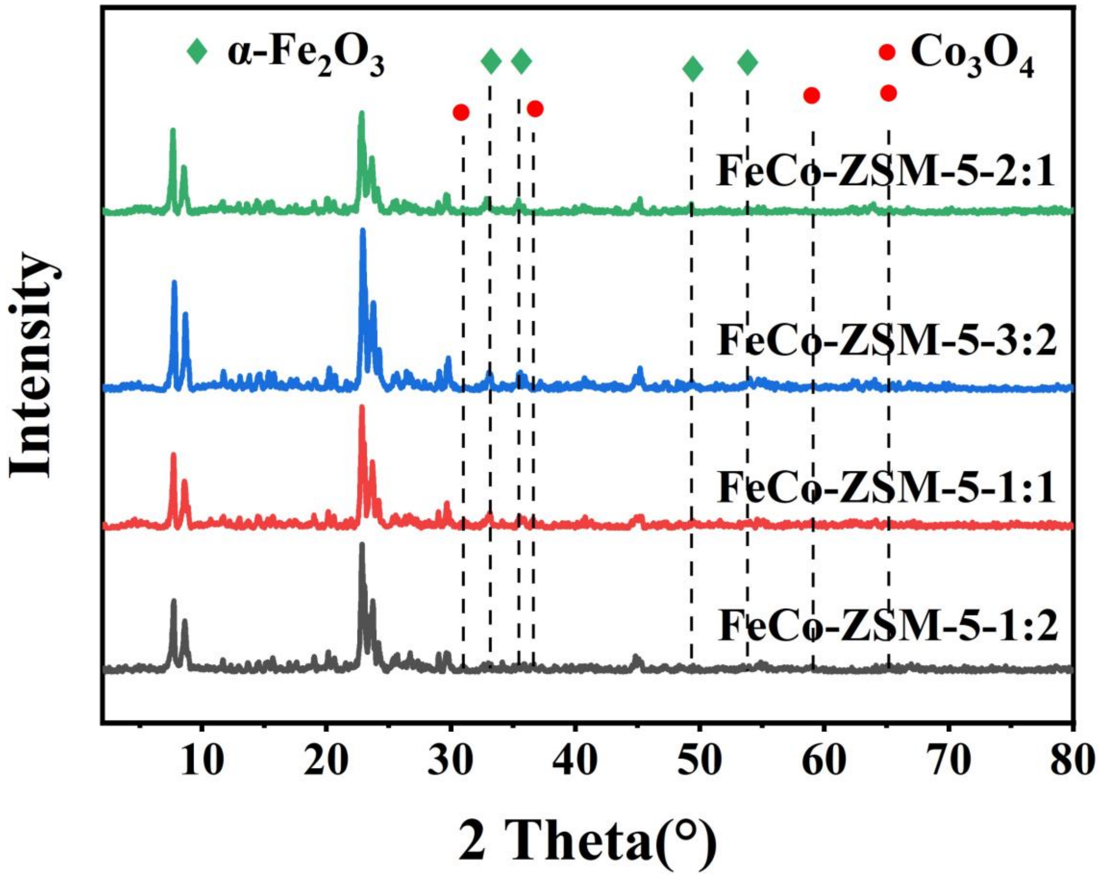

Figure S1. PXRD patterns of catalysts.

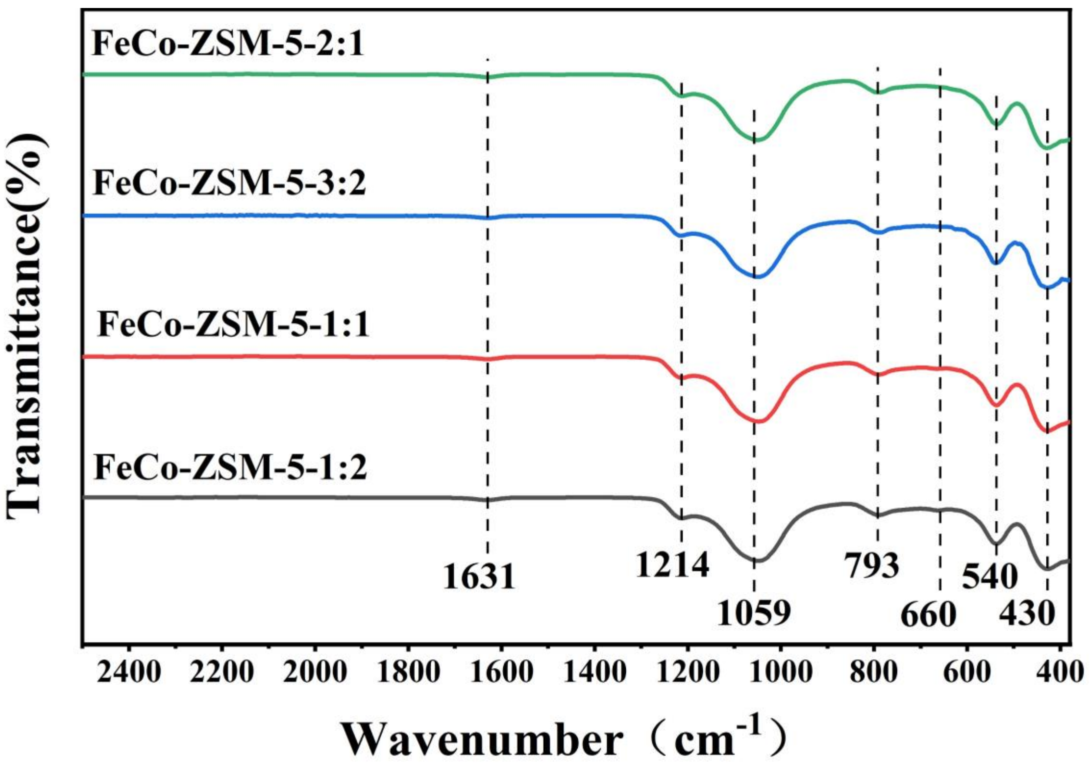

Figure S2. FTIR spectra of catalysts.

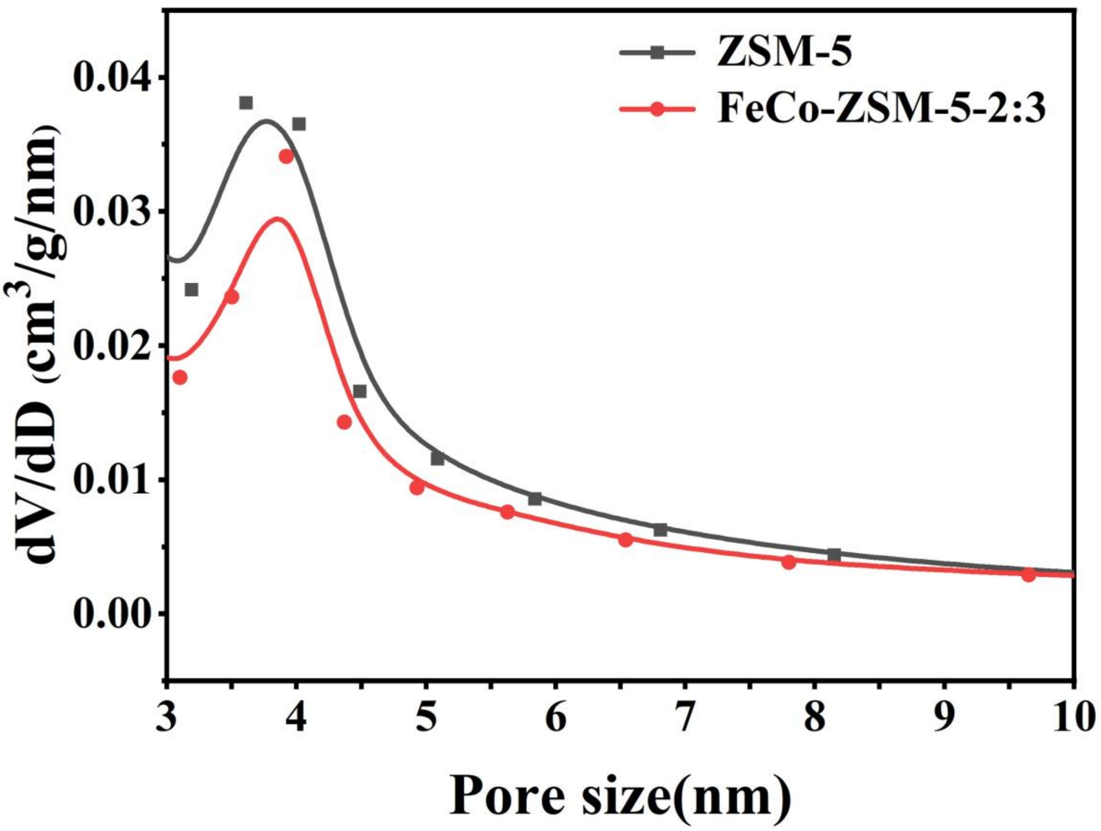

Figure S3. The pore size distribution of catalysts.

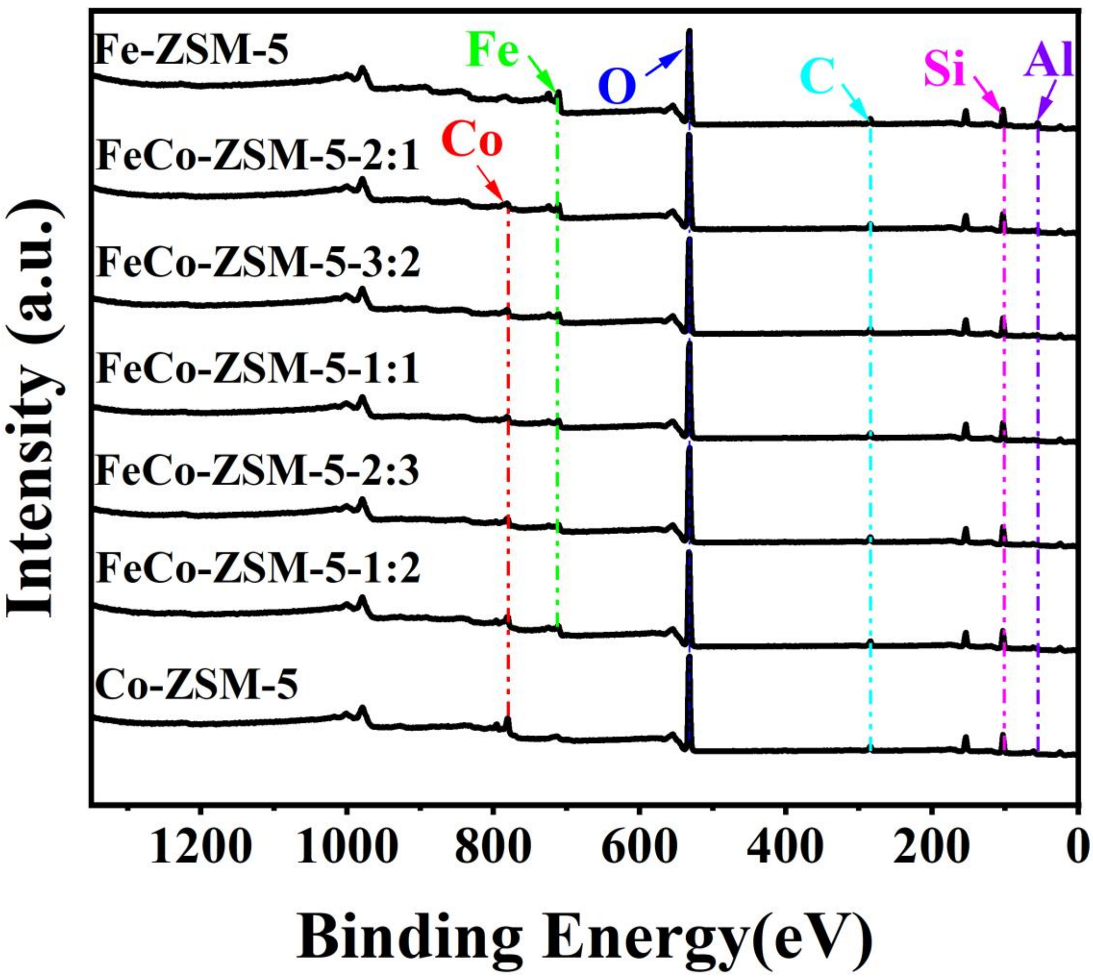

Figure S4. XPS survey of catalysts.

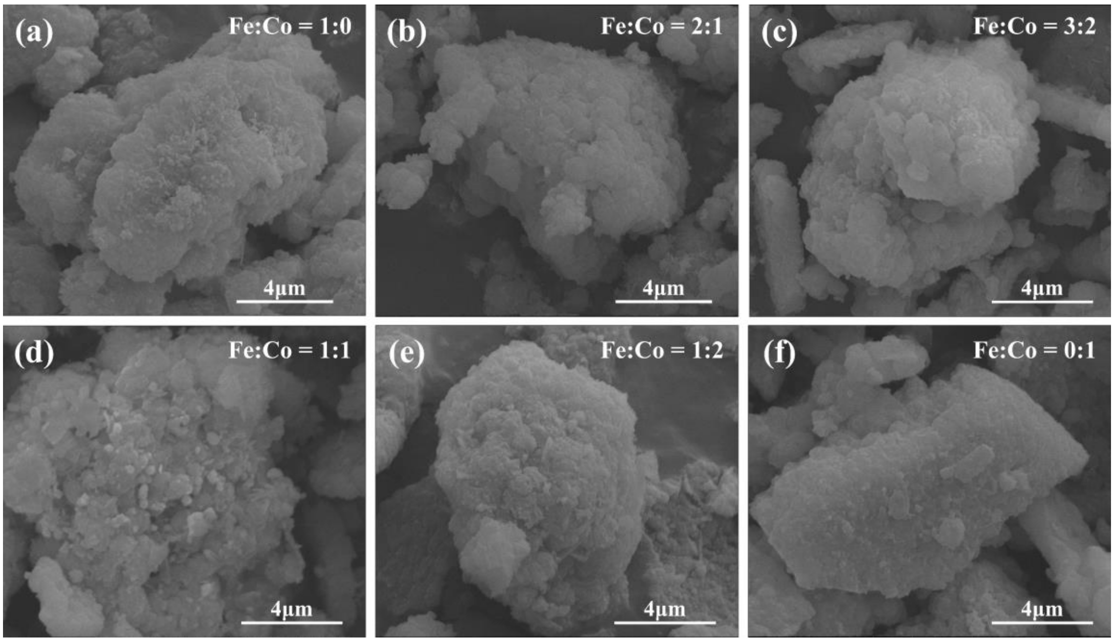

Figure S5. SEM images of catalysts: (a) Fe-ZSM-5, (b) FeCo-ZSM-5-2:1, (c) FeCo-ZSM-5-3:2, (d) FeCo-ZSM-5-1:1, (e) FeCo-ZSM-5-1:2, (f) Co-ZSM-5.

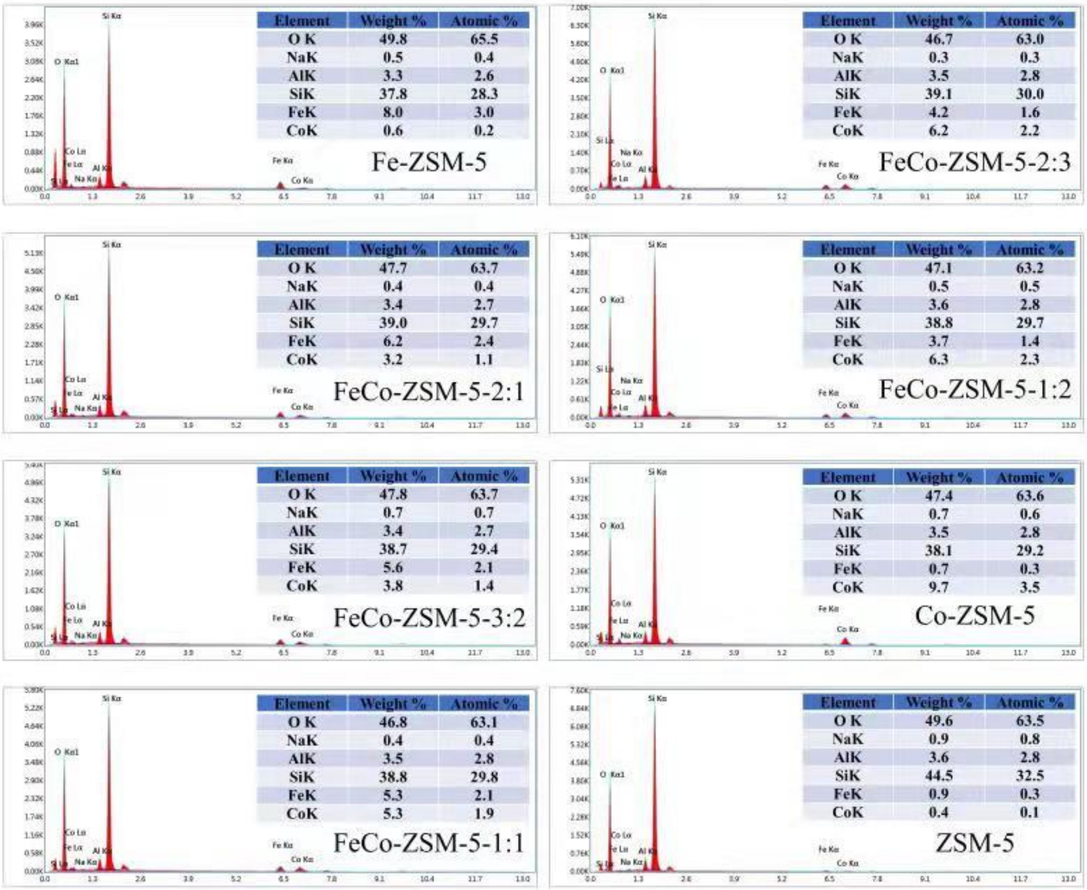

Figure S6. EDS element composition of FeCo-ZSM-5-*x*.

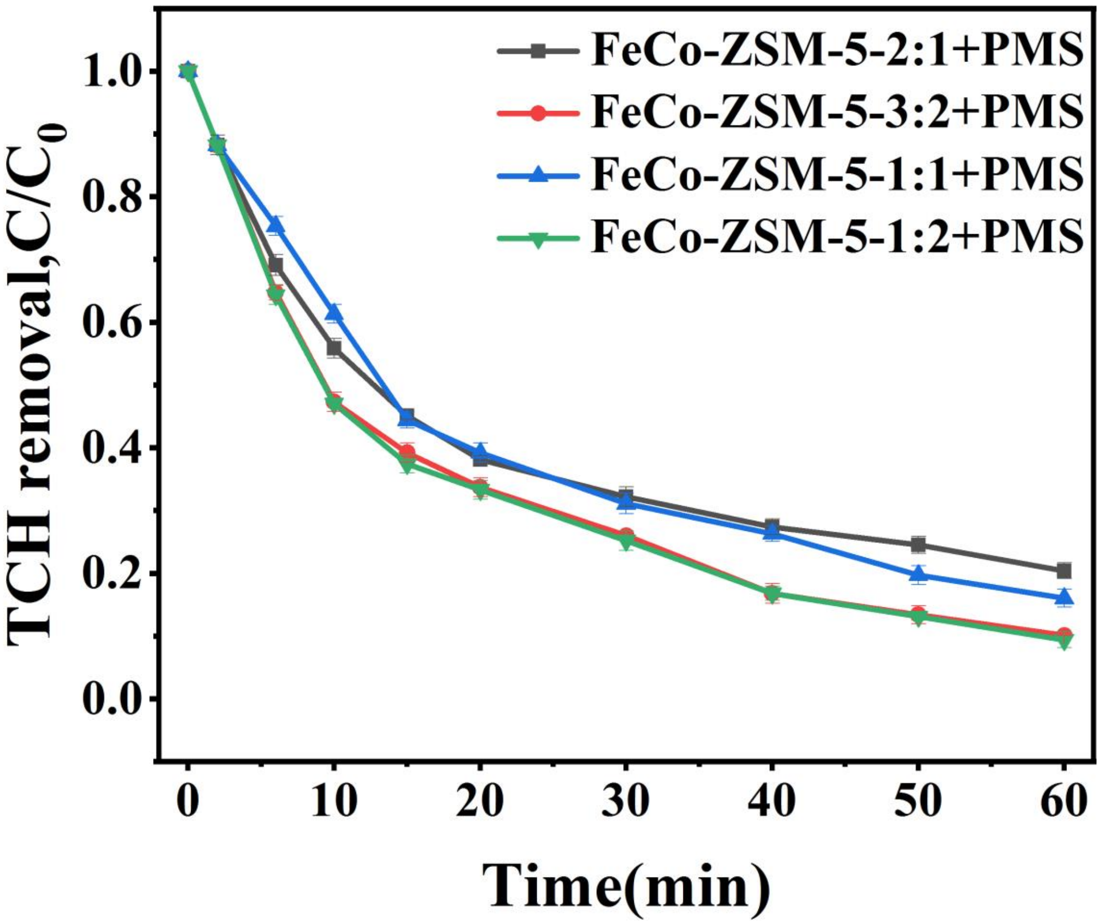

Figure S7. TCH removal by PMS activation on different catalysts. General conditions: [Pollutant] = 20 mg/L; [initial pH] = 7.0; [catalyst] = 0.5 g/L; [PMS] = 1 mM; and [temperature] = 25 ℃.

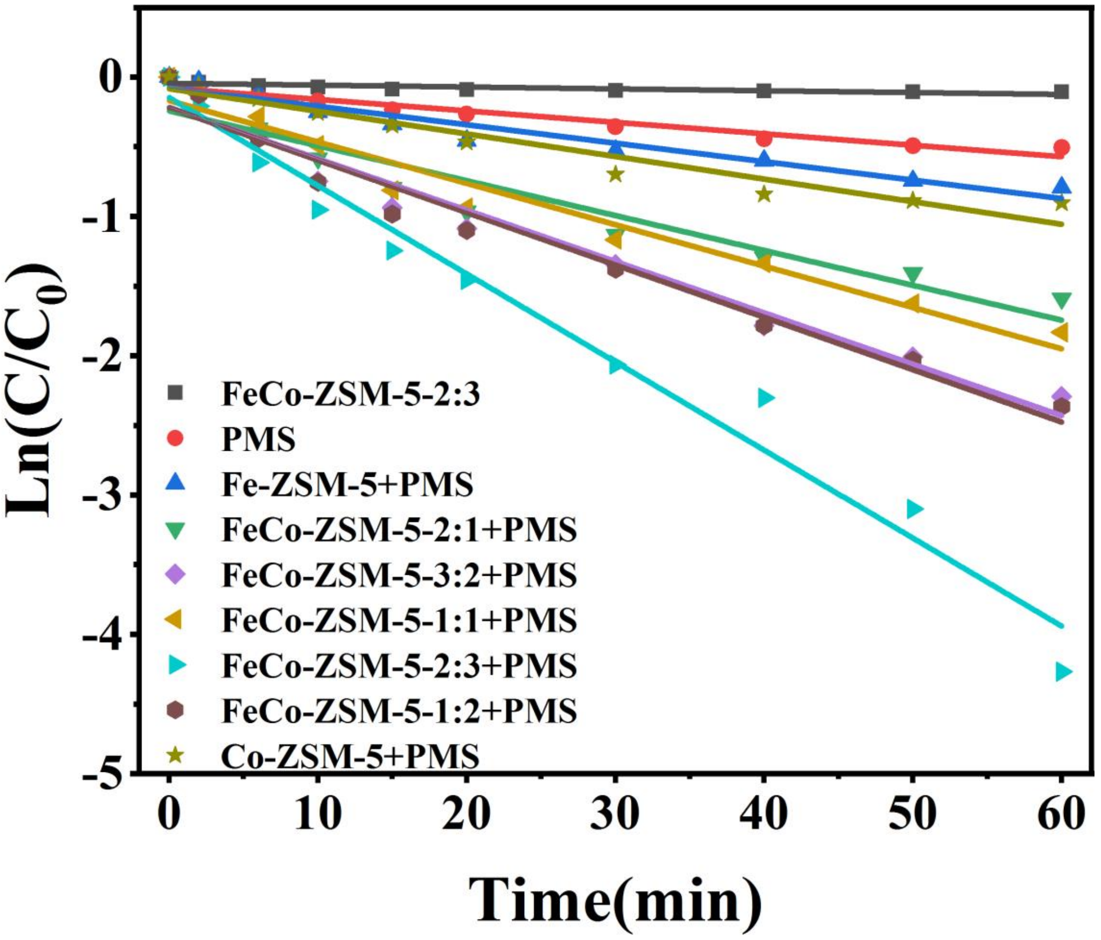

Figure S8. The liner fitted of different catalysts on TCH degradation in the presence of PMS. General conditions: [Pollutant] = 20 mg/L; [initial pH] = 7.0; [catalyst] = 0.5 g/L; [PMS] = 1 mM; and [temperature] = 25 ℃; (k was calculated as 0.0013, 0.0082, 0.0132, 0.0250, 0.0369, 0.0297, 0.0632, 0.0376, and 0.0162 min^{-1}, respectively)

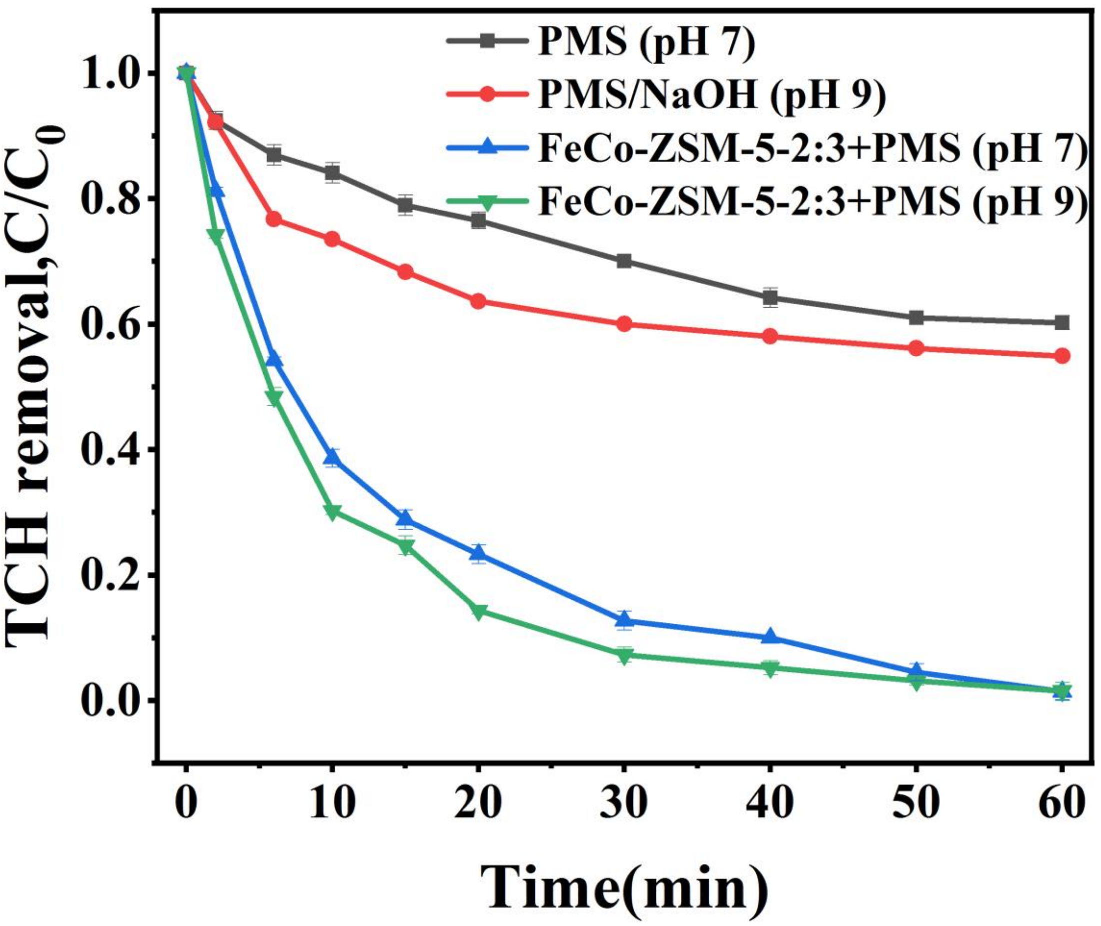

Figure S9. Control experiments of PMS/NaOH (pH 9). General conditions: [Pollutant] = 20 mg/L; [initial pH] = 7.0; [catalyst] = 0.5 g/L; [PMS] = 1 mM; and [temperature] = 25 ℃.

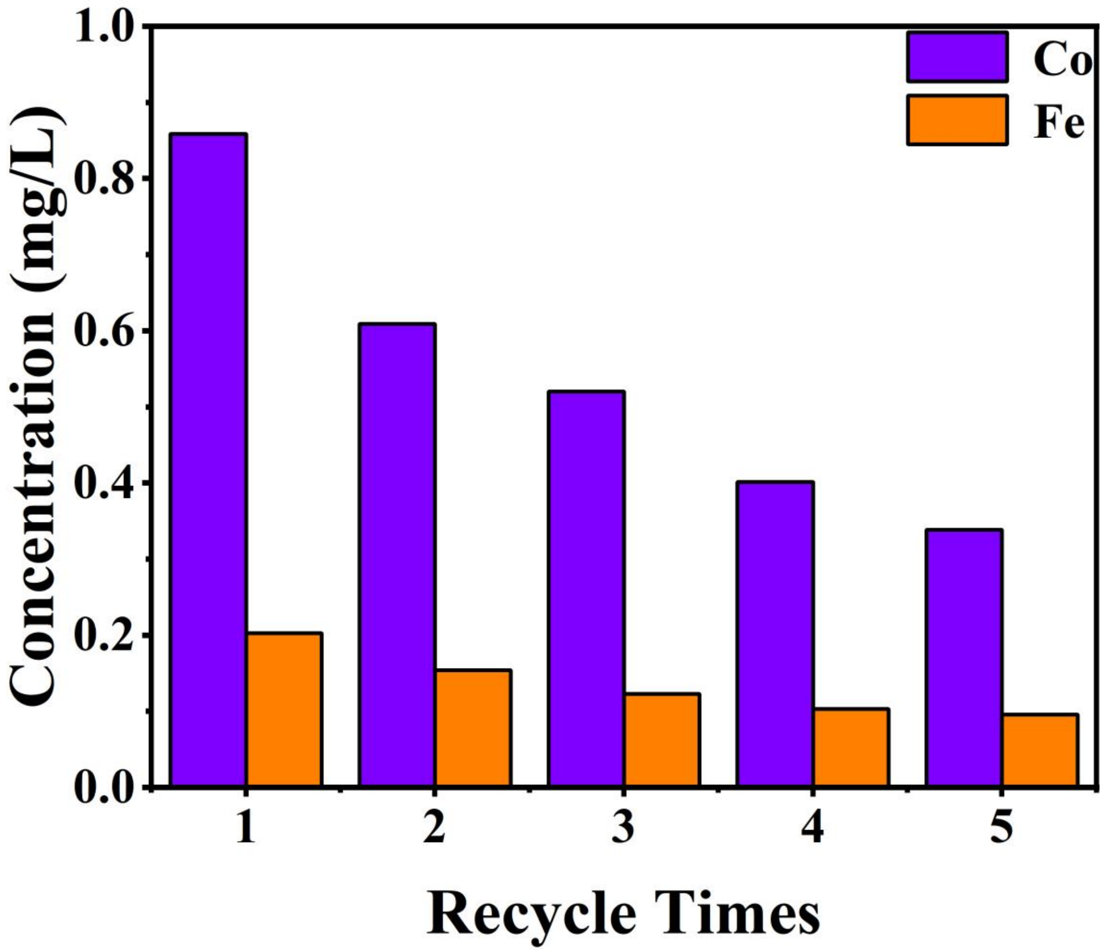

Figure S10. Metal leaching in different cycles.

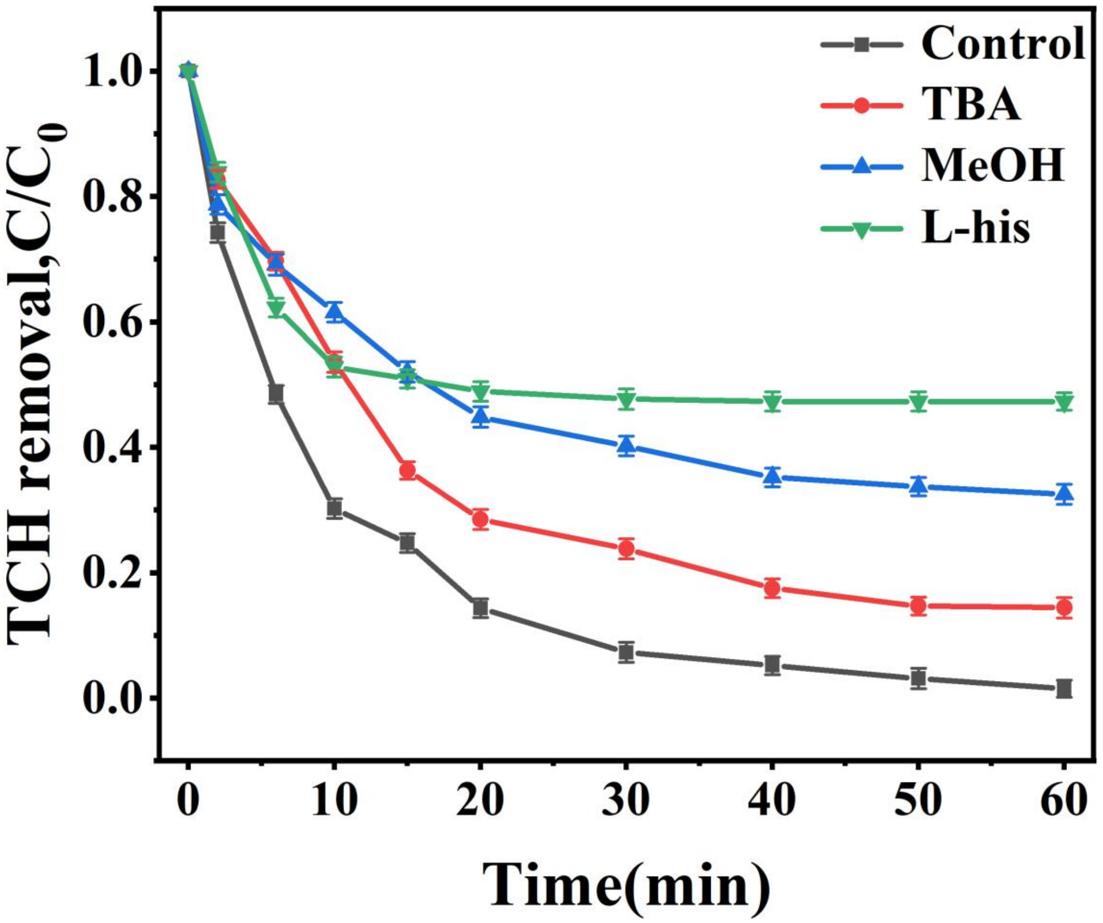

Figure S11 Quenching tests using different scavengers under alkaline condition.

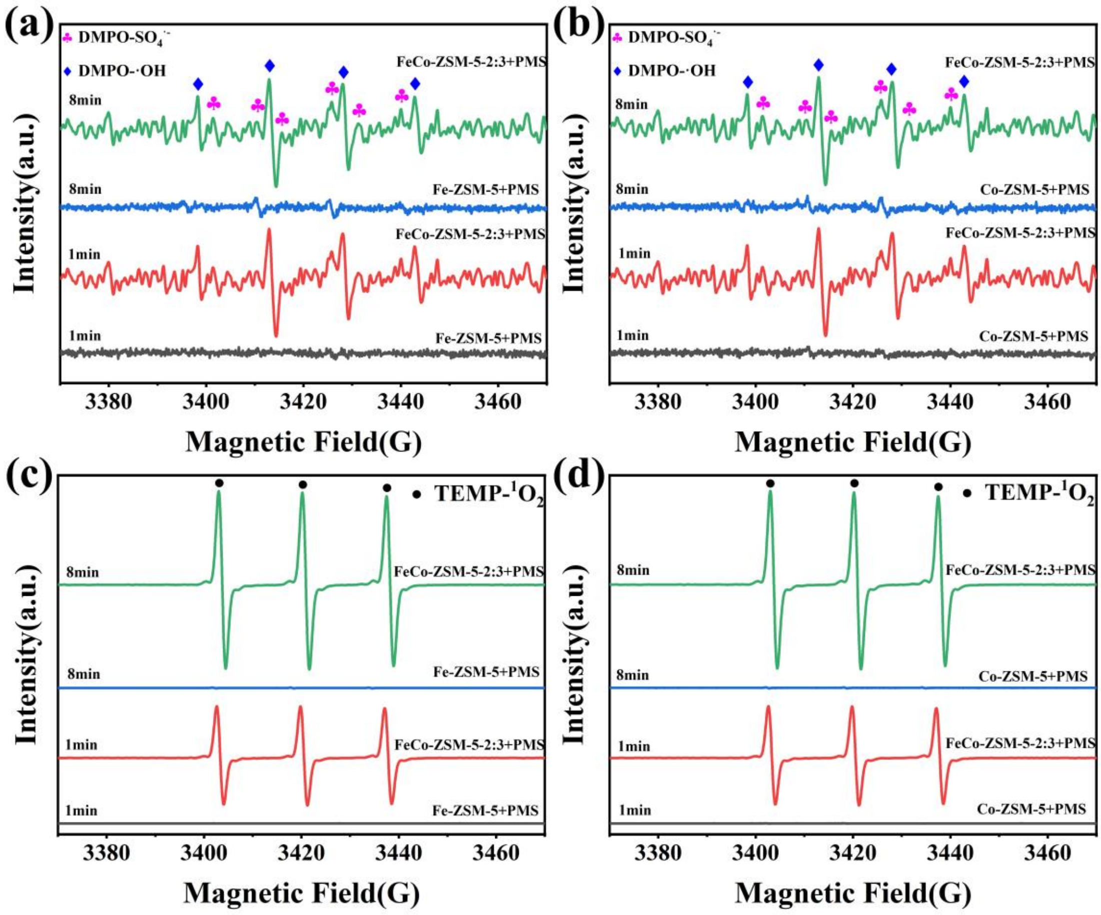

Figure S12. EPR spectra with DMPO in (a) FeCo-ZSM-5-2:3/PMS and Fe-ZSM-5/PMS systems and (b) FeCo-ZSM-5-2:3/PMS and Co-ZSM-5/PMS systems; EPR spectra with TEMP in (c) FeCo-ZSM-5-2:3/PMS and Fe-ZSM-5/PMS systems and (d) FeCo-ZSM-5-2:3/PMS and Co-ZSM-5/PMS systems.

Table S1. The final concentrations of FeCo-ZSM-5-*x* metals loaded by using ICP-MS.

m (Fe): m (Co)	1:0	2:1	3:2	1:1	2:3	1:2	0:1
Fe (Weight %)	8.9187	6.3211	5.6366	4.5849	3.6582	3.0947	0.0006
Co (Weight %)	0.0002	3.2287	3.7867	4.5214	5.4009	6.1837	9.5377

Table S2. The detailed fitting parameters for the XPS spectra.

Peak fitting data of Fe XPS									
Sample	Peak1		Peak2		Peak3		Peak4		χ^2
	Bending Energy(eV)	FWHM	Bending Energy(eV)	FWHM	Bending Energy(eV)	FWHM	Bending Energy(eV)	FWHM	
Fe-ZSM-5	710.30	1.10	723.90	1.78	711.36	2.54	724.96	3.17	1.17
FeCo-ZSM-5-2:1	710.44	1.80	724.04	2.39	711.92	3.10	725.52	4.06	0.93
FeCo-ZSM-5-1:1	710.49	2.09	724.09	2.86	712.13	3.07	725.73	4.46	1.12
FeCo-ZSM-5-2:3	710.32	1.97	723.92	2.38	711.76	2.23	725.36	2.85	1.34
Peak fitting data of Co XPS									
Sample	Peak1		Peak2		Peak3		Peak4		χ^2
	Bending Energy(eV)	FWHM	Bending Energy(eV)	FWHM	Bending Energy(eV)	FWHM	Bending Energy(eV)	FWHM	
FeCo-ZSM-5-1:1	780.55	2.47	796.25	3.33	782.71	2.84	798.41	4.35	1.40
FeCo-ZSM-5-2:3	780.36	2.19	796.06	2.70	782.36	2.63	798.06	3.81	1.26
FeCo-ZSM-5-1:2	780.34	1.86	796.04	2.44	782.10	2.55	797.80	3.12	1.51
Co-ZSM-5	780.05	1.44	795.75	2.17	781.90	2.62	797.50	4.54	1.89
Peak fitting data of O XPS									
Sample	Peak1		Peak2		Peak3		Peak4		χ^2
	Bending Energy(eV)	FWHM	Bending Energy(eV)	FWHM	Bending Energy(eV)	FWHM	Bending Energy(eV)	FWHM	
Fe-ZSM-5	530.25	1.70	531.74	1.86	533.14	2.32	534.76	1.46	1.95
FeCo-ZSM-5-2:3	330.21	1.49	531.48	1.54	532.88	1.86	534.31	1.84	2.52
Co-ZSM-5	530.47	1.05	531.49	1.50	533.05	1.76	534.40	1.83	2.37
ZSM-5	-	-	531.57	1.49	533.13	1.65	534.51	1.87	2.18

Table S3. EDS element composition of FeCo-ZSM-5-*x*.

m (Fe): m (Co)	1:0	2:1	3:2	1:1	2:3	1:2	0:1
Fe (Weight %)	8	6.2	5.6	5.3	4.2	3.7	0.7
Co (Weight %)	0.6	3.2	3.8	5.3	6.2	6.3	9.7

Table S4. Zeta potential at different pH.

pH	3	5	7	9
zeta potentia (mV)	-14.6	-34.0	-36.7	-37.2

Table S5. The coordinate data of Fe-ZSM-5, Co-ZSM-5 and FeCo-ZSM-5.

Co-ZSM-5 cluster model				Fe-ZSM-5 cluster model				FeCo-ZSM-5 cluster model			
**Atomic name**	**x(Å)**	**Y(Å)**	**Z (Å)**	**Atomic name**	**x(Å)**	**Y(Å)**	**Z (Å)**	**Atomic name**	**x(Å)**	**Y(Å)**	**Z (Å)**
Si1	13.29655	25.59862	13.82955	Si1	13.88175	24.69921	12.18449	Si1	13.88175	24.69921	12.18449
Si2	13.31941	22.4931	13.73035	Si2	13.90461	21.59369	12.08529	Si2	13.90461	21.59369	12.08529
O1	12.83897	26.43668	12.53971	O1	13.42416	25.53727	10.89465	O1	13.42416	25.53727	10.89465
O2	12.94631	24.04998	13.57857	O2	13.5315	23.15057	11.9335	O2	13.5315	23.15057	11.9335
O3	13.03774	21.7598	12.34354	O3	13.62294	20.86039	10.69848	O3	13.62294	20.86039	10.69848
Si3	16.38116	25.60124	13.65832	Si3	16.96636	24.70183	12.01326	Si3	16.96636	24.70183	12.01326
Si4	16.37242	22.49873	13.6369	Si4	16.95761	21.59932	11.99183	Si4	16.95761	21.59932	11.99183
O4	16.56006	26.23964	12.20609	O4	17.14526	25.34023	10.56103	O4	17.14526	25.34023	10.56103
O5	16.7807	24.04797	13.59522	O5	17.3659	23.14856	11.95016	O5	17.3659	23.14856	11.95016
O6	16.52031	21.88647	12.16912	O6	17.1055	20.98706	10.52406	O6	17.1055	20.98706	10.52406
O7	14.85854	25.7812	14.09899	O7	15.44373	24.88179	12.45392	O7	15.44373	24.88179	12.45392
O8	14.85655	22.32078	14.12708	O8	15.44174	21.42137	12.48202	O8	15.44174	21.42137	12.48202
Si5	13.35857	28.63016	22.38164	Si5	13.94376	27.73075	20.73658	Si5	13.94376	27.73075	20.73658
Si6	12.54875	26.39607	24.34045	Si6	13.13395	25.49666	22.69538	Si6	13.13395	25.49666	22.69538
Si7	12.53921	21.56838	24.21743	Si7	13.12441	20.66897	22.57237	Si7	13.12441	20.66897	22.57237
Si8	13.53071	24.01701	22.59728	Si8	14.11591	23.1176	20.95221	Si8	14.11591	23.1176	20.95221
O9	13.09937	27.74163	23.68508	O9	13.68456	26.84222	22.04001	O9	13.68456	26.84222	22.04001
O10	12.83897	26.43668	25.90871	O10	13.42416	25.53727	24.26365	O10	13.42416	25.53727	24.26365
O11	13.03774	21.7598	25.71254	O11	13.62294	20.86039	24.06748	O11	13.62294	20.86039	24.06748
O12	13.27429	22.58418	23.23517	O12	13.85948	21.68477	21.59011	O12	13.85948	21.68477	21.59011
O13	13.30609	25.12772	23.71608	O13	13.89129	24.22831	22.07102	O13	13.89129	24.22831	22.07102
O14	10.99234	26.24768	24.05196	O14	11.57753	25.34827	22.4069	O14	11.57753	25.34827	22.4069
O15	10.97047	21.81811	24.15783	O15	11.55567	20.9187	22.51277	O15	11.55567	20.9187	22.51277
O16	9.968637	28.45945	21.09334	O16	10.55383	27.56004	19.44828	O16	10.55383	27.56004	19.44828
O17	12.56267	28.02514	21.12718	O17	13.14786	27.12573	19.48212	O17	13.14786	27.12573	19.48212
O18	10.0	23.8	21.4	O18	10.5	22.9	19.8	O18	10.5	22.9	19.8

	0243	5092	5658		8762	5151	1152		8762	5151	1152
O19	12.5746	24.27719	21.35832	O19	13.15979	23.37778	19.71326	O19	13.15979	23.37778	19.71326
O20	12.8827	20.08086	23.75312	O20	13.46789	19.18145	22.10806	O20	13.46789	19.18145	22.10806
Al1	16.46902	28.66092	22.27669	Al1	17.05422	27.76151	20.63162	Al1	17.05422	27.76151	20.63162
Si9	17.36252	26.38541	24.21036	Si9	17.94772	25.486	22.56529	Si9	17.94772	25.486	22.56529
Si10	17.30567	21.54586	24.19391	Si10	17.89087	20.64645	22.54885	Si10	17.89087	20.64645	22.54885
Al2	16.59226	23.94321	22.34503	Al2	17.17746	23.04381	20.69997	Al2	17.17746	23.04381	20.69997
O21	16.8304	27.68533	23.48102	O21	17.41559	26.78592	21.83595	O21	17.41559	26.78592	21.83595
O22	16.56006	26.23964	25.57509	O22	17.14526	25.34023	23.93003	O22	17.14526	25.34023	23.93003
O23	16.52031	21.88647	25.53812	O23	17.1055	20.98706	23.89306	O23	17.1055	20.98706	23.89306
O24	16.87413	22.53593	23.02462	O24	17.45932	21.63652	21.37956	O24	17.45932	21.63652	21.37956
O25	17.14248	25.09957	23.29689	O25	17.72767	24.20016	21.65182	O25	17.72767	24.20016	21.65182
O26	18.91358	26.51912	24.52484	O26	19.49877	25.61971	22.87978	O26	19.49877	25.61971	22.87978
O27	18.86587	21.67736	24.47326	O27	19.45106	20.77795	22.8282	O27	19.45106	20.77795	22.8282
O28	19.79614	27.99498	21.30328	O28	20.38134	27.09557	19.65822	O28	20.38134	27.09557	19.65822
O29	17.21404	28.19404	20.94043	O29	17.79923	27.29463	19.29537	O29	17.79923	27.29463	19.29537
O30	17.34324	24.02384	20.93625	O30	17.92844	23.12443	19.29118	O30	17.92844	23.12443	19.29118
O31	16.94569	20.06075	23.73502	O31	17.53088	19.16134	22.08995	O31	17.53088	19.16134	22.08995
O32	14.90624	28.63036	22.00084	O32	15.49144	27.73095	20.35578	O32	15.49144	27.73095	20.35578
O33	15.03744	24.10226	22.07551	O33	15.62263	23.20285	20.43045	O33	15.62263	23.20285	20.43045
Al3	13.34744	18.60742	24.16613	Al3	13.93263	17.70801	22.52107	Al3	13.93263	17.70801	22.52107
Si11	12.45394	16.33191	22.23246	Si11	13.03913	15.4325	20.5874	Si11	13.03913	15.4325	20.5874
Si12	13.4353	15.54774	19.4155	Si12	14.02049	14.64833	17.77044	Si12	14.02049	14.64833	17.77044
O34	12.98606	17.63183	22.9618	O34	13.57126	16.73242	21.31674	O34	13.57126	16.73242	21.31674
O35	13.2564	16.18614	20.86773	O35	13.84159	15.28673	19.22267	O35	13.84159	15.28673	19.22267
O36	13.03576	13.99447	19.4786	O36	13.62095	13.09506	17.83353	O36	13.62095	13.09506	17.83353
O37	12.67398	15.04607	23.14593	O37	13.25918	14.14666	21.50087	O37	13.25918	14.14666	21.50087
O38	10.90289	16.46562	21.91798	O38	11.48808	15.56621	20.27292	O38	11.48808	15.56621	20.27292
O39	12.6	18.1	25.5	O39	13.1	17.2	23.8	O39	13.1	17.2	23.8

	0242	4054	0239		8762	4113	5733		8762	4113	5733
O40	14.91022	18.57686	24.44198	O40	15.49541	17.67745	22.79691	O40	15.49541	17.67745	22.79691
O41	14.95792	15.7277	18.97483	O41	15.54312	14.82829	17.32977	O41	15.54312	14.82829	17.32977
Si13	16.45789	18.57666	24.06118	Si13	17.04309	17.67725	22.41611	Si13	17.04309	17.67725	22.41611
Si14	17.26771	16.34257	22.10237	Si14	17.8529	15.44316	20.45731	Si14	17.8529	15.44316	20.45731
Si15	16.51991	15.54512	19.24427	Si15	17.1051	14.64571	17.59921	Si15	17.1051	14.64571	17.59921
O42	16.7171	17.68813	22.75774	O42	17.30229	16.78872	21.11268	O42	17.30229	16.78872	21.11268
O43	16.97749	16.38318	20.53411	O43	17.56269	15.48377	18.88904	O43	17.56269	15.48377	18.88904
O44	16.87015	13.99648	19.49525	O44	17.45535	13.09707	17.85019	O44	17.45535	13.09707	17.85019
O45	16.51037	15.07422	22.72674	O45	17.09556	14.17481	21.08167	O45	17.09556	14.17481	21.08167
O46	18.82413	16.19418	22.39086	O46	19.40932	15.29477	20.74579	O46	19.40932	15.29477	20.74579
O47	17.25379	17.97164	25.31564	O47	17.83899	17.07223	23.67058	O47	17.83899	17.07223	23.67058
O48	12.87077	30.11425	22.7078	O48	13.45597	29.21484	21.06274	O48	13.45597	29.21484	21.06274
O49	16.93376	30.13436	22.6897	O49	17.51896	29.23495	21.04464	O49	17.51896	29.23495	21.04464
Si16	19.32027	18.56318	15.25497	Si16	19.90547	17.66378	13.6099	Si16	19.90547	17.66378	13.6099
Si17	18.76747	16.17488	17.33817	Si17	19.35267	15.27547	15.69311	Si17	19.35267	15.27547	15.69311
Si18	10.47254	18.68021	15.6321	Si18	11.05773	17.7808	13.98703	Si18	11.05773	17.7808	13.98703
Si19	11.17561	16.33955	17.48203	Si19	11.7608	15.44014	15.83697	Si19	11.7608	15.44014	15.83697
O50	18.54385	18.03196	14.20027	O50	19.12905	17.13255	12.55521	O50	19.12905	17.13255	12.55521
O51	18.99706	17.63384	16.74712	O51	19.58226	16.73443	15.10205	O51	19.58226	16.73443	15.10205
O52	18.92948	15.11443	16.15299	O52	19.51467	14.21502	14.50792	O52	19.51467	14.21502	14.50792
O53	17.29752	16.07957	17.95683	O53	17.88272	15.18016	16.31177	O53	17.88272	15.18016	16.31177
O54	19.82596	15.88654	18.49545	O54	20.41115	14.98713	16.85038	O54	20.41115	14.98713	16.85038
O55	18.85394	20.04266	15.87713	O55	19.43914	19.14325	14.23207	O55	19.43914	19.14325	14.23207
O56	10.97643	18.47431	14.13503	O56	11.56163	17.5749	12.48996	O56	11.56163	17.5749	12.48996
O57	11.22888	17.6821	16.61603	O57	11.81407	16.78269	14.97097	O57	11.81407	16.78269	14.97097
O58	11.12949	15.0702	16.51559	O58	11.71469	14.17079	14.87053	O58	11.71469	14.17079	14.87053
O59	8.907171	18.39589	15.71671	O59	9.492366	17.49648	14.07165	O59	9.492366	17.49648	14.07165
O60	12.5	16.2	18.3	O60	13.0	15.3	16.7	O60	13.0	15.3	16.7

	0105	8667	6586		8624	8726	208		8624	8726	208
O61	9.875212	16.36308	18.41796	O61	10.46041	15.46367	16.7729	O61	10.46041	15.46367	16.7729
O62	10.78163	20.18542	16.05575	O62	11.36683	19.28601	14.41069	O62	11.36683	19.28601	14.41069
Si20	11.04123	21.7043	15.64764	Si20	11.62643	20.80489	14.00258	Si20	11.62643	20.80489	14.00258
Si21	10.55304	23.85334	17.74319	Si21	11.13823	22.95393	16.09813	Si21	11.13823	22.95393	16.09813
Si22	11.18097	24.53858	20.63745	Si22	11.76617	23.63917	18.99239	Si22	11.76617	23.63917	18.99239
Si23	11.17779	27.66824	20.41396	Si23	11.76299	26.76883	18.7689	Si23	11.76299	26.76883	18.7689
Si24	10.49619	28.61668	17.81885	Si24	11.08138	27.71728	16.17379	Si24	11.08138	27.71728	16.17379
Si25	11.04899	26.22838	15.73565	Si25	11.63418	25.32897	14.09059	Si25	11.63418	25.32897	14.09059
Si26	18.74402	21.54726	15.36157	Si26	19.32921	20.64785	13.71651	Si26	19.32921	20.64785	13.71651
Si27	19.34194	23.87968	17.31207	Si27	19.92713	22.98027	15.667	Si27	19.92713	22.98027	15.667
Si28	18.66292	24.59006	20.2283	Si28	19.24811	23.69065	18.58324	Si28	19.24811	23.69065	18.58324
Si29	18.59812	27.71649	20.28788	Si29	19.18331	26.81708	18.64282	Si29	19.18331	26.81708	18.64282
Si30	19.34393	28.73371	17.44172	Si30	19.92912	27.8343	15.79666	Si30	19.92912	27.8343	15.79666
Si31	18.64085	26.39305	15.59179	Si31	19.22605	25.49364	13.94673	Si31	19.22605	25.49364	13.94673
O63	11.10763	22.56206	16.99045	O63	11.69282	21.66266	15.34538	O63	11.69282	21.66266	15.34538
O64	11.25472	23.91527	19.16787	O64	11.83992	23.01586	17.52281	O64	11.83992	23.01586	17.52281
O65	10.87705	26.10291	20.54796	O65	11.46224	25.2035	18.9029	O65	11.46224	25.2035	18.9029
O66	11.27261	28.08546	18.87355	O66	11.85781	27.18605	17.22848	O66	11.85781	27.18605	17.22848
O67	10.8194	27.68734	16.3267	O67	11.4046	26.78793	14.68164	O67	11.4046	26.78793	14.68164
O68	10.88698	25.16793	16.92083	O68	11.47218	24.26852	15.27577	O68	11.47218	24.26852	15.27577
O69	8.974755	23.74838	17.95657	O69	9.55995	22.84897	16.3115	O69	9.55995	22.84897	16.3115
O70	8.927049	28.59014	17.90498	O70	9.512244	27.69073	16.25992	O70	9.512244	27.69073	16.25992
O71	12.42949	21.82615	14.8667	O71	13.01468	20.92674	13.22163	O71	13.01468	20.92674	13.22163
O72	9.857322	22.27252	14.73501	O72	10.44252	21.37311	13.08995	O72	10.44252	21.37311	13.08995
O73	12.51894	26.13307	15.11699	O73	13.10413	25.23366	13.47193	O73	13.10413	25.23366	13.47193
O74	9.990502	25.94004	14.57837	O74	10.5757	25.04063	12.93331	O74	10.5757	25.04063	12.93331
O75	10.96252	30.09616	17.19669	O75	11.54771	29.19675	15.55163	O75	11.54771	29.19675	15.55163
O76	18.8	22.5	16.6	O76	19.4	21.6	14.9	O76	19.4	21.6	14.9

	8972	2185	1334		7492	2244	6828		7492	2244	6828
O77	18.844	23.84087	18.82511	O77	19.4292	22.94146	17.18005	O77	19.4292	22.94146	17.18005
O78	18.52199	26.1572	19.96257	O78	19.10718	25.25779	18.31751	O78	19.10718	25.25779	18.31751
O79	18.84003	28.52781	18.93879	O79	19.42522	27.6284	17.29373	O79	19.42522	27.6284	17.29373
O80	18.58758	27.7356	16.45779	O80	19.17278	26.83619	14.81272	O80	19.17278	26.83619	14.81272
O81	18.68697	25.1237	16.55823	O81	19.27217	24.22429	14.91317	O81	19.27217	24.22429	14.91317
O82	17.32138	21.74371	14.65708	O82	17.90657	20.8443	13.01202	O82	17.90657	20.8443	13.01202
O83	17.31541	26.34017	14.70796	O83	17.90061	25.44076	13.06289	O83	17.90061	25.44076	13.06289
O84	19.03483	30.23892	17.01806	O84	19.62002	29.33951	15.373	O84	19.62002	29.33951	15.373
O85	19.92932	24.32746	21.14665	O85	20.51452	23.42805	19.50158	O85	20.51452	23.42805	19.50158
O86	20.93116	24.01982	17.25124	O86	21.51635	23.12041	15.60617	O86	21.51635	23.12041	15.60617
O87	19.94125	26.41658	14.65586	O87	20.52644	25.51717	13.0108	O87	20.52644	25.51717	13.0108
O88	20.88941	18.53664	15.16883	O88	21.47461	17.63723	13.52377	O88	21.47461	17.63723	13.52377
O89	19.90746	21.80805	14.29262	O89	20.49265	20.90864	12.64755	O89	20.49265	20.90864	12.64755
H1	13.54071	26.76269	11.95449	H1	14.10168	25.72143	10.22572	H1	14.10367	25.75879	10.24011
H2	12.85207	22.30965	11.56951	H2	12.97574	21.25046	10.09368	H2	14.35696	20.79352	10.06845
H3	17.39948	26.68036	12.00878	H3	18.04034	25.55052	10.257	H3	17.99237	25.75775	10.34646
H4	17.24239	22.24263	11.63003	H4	17.97124	21.06174	10.09569	H4	17.91063	21.19933	10.02867
H5	12.75459	27.29954	26.34112	H5	14.35611	25.63527	24.52547	H5	13.09478	26.29981	24.7607
H6	12.94003	20.98238	26.28753	H6	13.66956	20.03506	24.57682	H6	14.58597	20.84917	24.21532
H7	10.64338	26.16298	23.15199	H7	11.1251	24.53453	22.67709	H7	11.15903	24.48793	22.55951
H8	10.58511	22.37618	24.85015	H8	11.01368	20.12215	22.62875	H8	10.99627	20.22459	22.89322
H9	10.18838	29.26502	21.58833	H9	10.69969	28.02001	20.28894	H9	10.2352	27.20342	20.29361
H10	10.2101	23.12815	22.07159	H10	10.79962	22.29414	20.4985	H10	10.80475	22.23358	20.43004
H11	15.59241	26.17107	25.53376	H11	17.1162	24.4497	24.31522	H11	16.22308	25.63599	23.91747
H12	15.55381	21.99739	25.52977	H12	16.13341	21.02498	23.86032	H12	17.2777	21.87707	24.2444
H13	19.17875	27.03427	25.30108	H13	19.73847	26.19269	23.62243	H13	19.7351	26.27466	23.55305
H14	19.22538	22.57246	24.37325	H14	19.7297	21.51064	23.39694	H14	19.97358	21.18563	22.12179
H15	19.6	28.5	22.0	H15	20.3	26.5	20.4	H15	20.1	27.0	20.6

	0568	8709	4543		9848	7636	8217		8382	3434	0889
H16	12.8669	13.52842	18.64535	H16	13.52642	12.62725	16.99001	H16	13.40246	12.64633	17.00215
H17	13.57792	14.7215	23.286	H17	13.31188	14.3255	22.45218	H17	13.33912	14.30708	22.45609
H18	10.30543	16.76192	22.62149	H18	10.91179	14.91572	20.69895	H18	10.88772	14.99577	20.77432
H19	12.80516	17.44492	26.13444	H19	13.57057	16.80057	24.62419	H19	13.31098	17.56293	24.7545
H20	17.59578	13.61693	18.97831	H20	17.38032	12.78145	18.76564	H20	17.37828	12.76181	18.7579
H21	17.03657	14.47896	23.28083	H21	16.12625	14.2184	21.14514	H21	16.1489	14.23726	21.28476
H22	19.26897	16.81068	22.99121	H22	19.69903	15.49617	21.64908	H22	19.70215	15.42581	21.66137
H23	17.7717	18.58872	25.8547	H23	18.06331	17.68931	24.38211	H23	17.30654	16.99987	24.47612
H24	13.40358	30.8149	22.30099	H24	14.07081	29.93172	20.84279	H24	14.10014	29.76748	21.53414
H25	16.95732	30.58389	23.54155	H25	18.30925	29.53468	21.50182	H25	17.88057	29.95767	20.52545
H26	18.01382	18.28667	13.44091	H26	18.20035	17.07498	12.31733	H26	19.06902	16.29144	12.09266
H27	19.82768	14.90688	15.85765	H27	19.44274	13.27751	14.74492	H27	19.46156	13.27302	14.73133
H28	19.90102	16.52706	19.2185	H28	20.26634	15.36964	17.72977	H28	20.31804	15.42232	17.71109
H29	11.34514	19.22841	13.65266	H29	12.22356	18.18366	12.13101	H29	12.28881	18.11667	12.14841
H30	11.2442	15.22359	15.56605	H30	11.88827	14.31834	13.92945	H30	10.94555	14.05776	14.29266
H31	8.535753	18.16125	16.58061	H31	9.17304	17.01283	14.84927	H31	8.922	18.00061	13.47182
H32	9.984506	16.41694	19.38172	H32	10.6095	15.57717	17.72797	H32	10.53588	15.70471	17.70962
H33	20.80322	28.50727	17.32343	H33	21.38449	27.55041	15.74971	H33	21.38959	27.6204	15.68334
H34	8.40179	23.9789	17.20958	H34	9.20897	22.09578	16.80912	H34	9.245598	22.26079	17.01367
H35	8.473711	28.62446	18.76124	H35	9.035663	27.82917	17.09333	H35	9.04362	27.58301	17.10121
H36	9.970121	23.14276	14.31906	H36	10.45278	22.30762	12.82484	H36	10.40247	22.32047	12.87897
H37	9.570065	26.71496	14.17761	H37	9.682498	25.39811	13.05079	H37	10.50806	25.67809	12.20729
H38	11.75916	30.01384	16.64818	H38	11.19701	29.31581	14.65247	H38	11.77814	29.07926	14.61615
H39	18.35746	30.71569	17.52052	H39	20.01084	30.04245	15.91234	H39	18.82066	29.74359	15.7438
H40	20.11136	25.00841	21.81575	H40	20.44263	22.81907	20.25338	H40	20.58929	23.97506	20.30175
H41	21.40126	24.01043	18.09877	H41	22.03465	22.76071	16.34141	H41	22.00751	23.06479	16.43969
H42	20.75998	26.06215	15.0342	H42	21.30581	25.03082	13.32082	H42	21.33977	25.12654	13.36511
H43	21.3	18.5	16.0	H43	21.9	16.9	14.0	H43	21.9	17.4	14.3

	365	9481	2617		0661	1876	1023		2154	6244	6592
H44	20.8<br>1675	21.8<br>361	14.6<br>2516	H44	21.3<br>925	20.6<br>6566	12.9<br>1462	H44	21.4<br>0087	20.7<br>1997	12.9<br>2891
Co1	14.6<br>2661	18.5<br>0269	21.8<br>3219	Fe1	16.0<br>4323	23.7<br>2371	18.3<br>3901	Fe1	16.0<br>1254	23.5<br>9293	18.3<br>0727
O90	14.9<br>2141	18.6<br>2851	19.8<br>3236	Fe2	15.8<br>4872	27.0<br>5784	18.2<br>2763	Fe2	15.9<br>7491	26.9<br>364	18.1<br>2968
O91	14.8<br>6945	20.5<br>4627	21.7<br>0316	O90	16.2<br>4492	26.7<br>3005	16.6<br>3211	O90	16.3<br>7786	26.8<br>7697	16.5<br>3436
H45	14.8<br>8622	17.9<br>3568	19.1<br>4096	O91	15.4<br>0302	25.3<br>9935	18.6<br>7326	O91	15.2<br>7635	25.2<br>8829	18.3<br>1717
H46	14.1<br>6874	20.9<br>9032	22.2<br>4189	O92	16.5<br>6184	24.2<br>2359	16.4<br>0833	O92	16.5<br>2814	23.3<br>6775	16.2<br>3642
H47	15.7<br>0321	20.9<br>7958	22.0<br>2196	O93	14.8<br>5651	22.1<br>2686	17.5<br>363	O93	15.2<br>9903	21.5<br>9062	17.9<br>9873
H48	14.7<br>8183	19.5<br>0277	19.4<br>2144	O94	14.3<br>6246	29.0<br>6402	15.4<br>9334	O94	14.3<br>5263	24.6<br>1905	15.8<br>7638
				O95	15.4<br>1407	29.0<br>1516	17.8<br>3332	O95	15.5<br>3605	29.0<br>6156	17.8<br>4167
				H45	16.0<br>7831	23.8<br>3281	15.6<br>5823	H45	15.6<br>9036	23.8<br>5833	15.8<br>8315
				H46	13.8<br>8567	22.2<br>9164	17.5<br>6637	H46	15.7<br>0315	21.1<br>8081	17.2<br>1258
				H47	14.9<br>8347	21.2<br>4978	17.9<br>4882	H47	15.2<br>9163	20.9<br>0442	18.7<br>1086
				H48	16.4<br>4618	25.2<br>6352	16.3<br>685	H48	17.2<br>7506	23.9<br>4381	15.9<br>815
				H49	14.5<br>4179	28.2<br>0577	15.0<br>6584	H49	14.5<br>6725	25.1<br>3127	16.7<br>0375
				H50	13.3<br>7536	29.1<br>7184	15.4<br>7991	H50	14.3<br>3329	25.2<br>6403	15.1<br>4591
				H51	15.0<br>24	29.0<br>5999	16.8<br>7004	H51	14.8<br>9635	29.4<br>2774	18.4<br>8215
				H52	16.2<br>3313	29.5<br>477	17.8<br>3258	H52	15.1<br>0272	29.0<br>8006	16.9<br>6697
								Co1	14.6<br>6206	17.5<br>1452	19.6<br>2395
								O96	14.7<br>5897	17.9<br>5254	17.5<br>5041
								O97	15.2<br>8672	19.4<br>8262	20.0<br>2995
								H53	14.6<br>1877	19.6<br>5993	20.7<br>5825
								H54	16.1<br>5374	19.5<br>8596	20.4<br>9561
								H55	13.9<br>6265	17.6<br>6825	17.0<br>5636
								H56	15.4<br>8513	17.4<br>5464	17.1<br>2249

Text S1. Possible degradation pathway of TCH.

TCH is easily attacked by active radicals generated by PMS activation to generate intermediate products due to highly reactive functional groups such as phenolic hydroxyl groups in the TCH molecule. LC-MS is employed to identify the possible reaction intermediates in the solution during TCH degradation (**Figure S13**), and 11 representative intermediate structures are selected to infer two probable degradation routes as shown in **Figure S14**.

In pathway I, intermediate T1 (*m/z* 487) was generated through hydration and methyl oxidation reaction firstly. And then, T2 (*m/z* 340) could be formed by the removal of aldehyde group, hydroxyl group and amide bond. Meanwhile, T3 (*m/z* 255) and T4 (*m/z* 201) was produced from the reaction of ring opening and dehydration reactions. For pathway II, intermediate T5 (*m/z* 437) was formed via the demethylation and hydration of the TCH. Then, T6 (*m/z* 349) could be produced through ring-opening and dehydration, and T7 (*m/z* 223) was derived from continued ring-opening and dealkylation. The intermediates P1~P4 were obtained through ring-opening reaction, decarboxylation reaction, and the final products carbon dioxide and water were produced through further oxidation reactions to achieve complete degradation of TCH. It has been reported that $SO_4^{\bullet-}$ could participate in dehydroxylation, deamination and dedimethylamination reactions [1]. And $^1O_2$ took part in the reaction of methylene oxidation [2]. The catalyst activated PMS to produce more $SO_4^{\bullet-}$ and $^1O_2$. In the degradation pathway, T1, T2, T5 and T6 could be obtained with the participation of $SO_4^{\bullet-}$, and intermediates T3, T4 and T7 were generated through the participation of $^1O_2$.

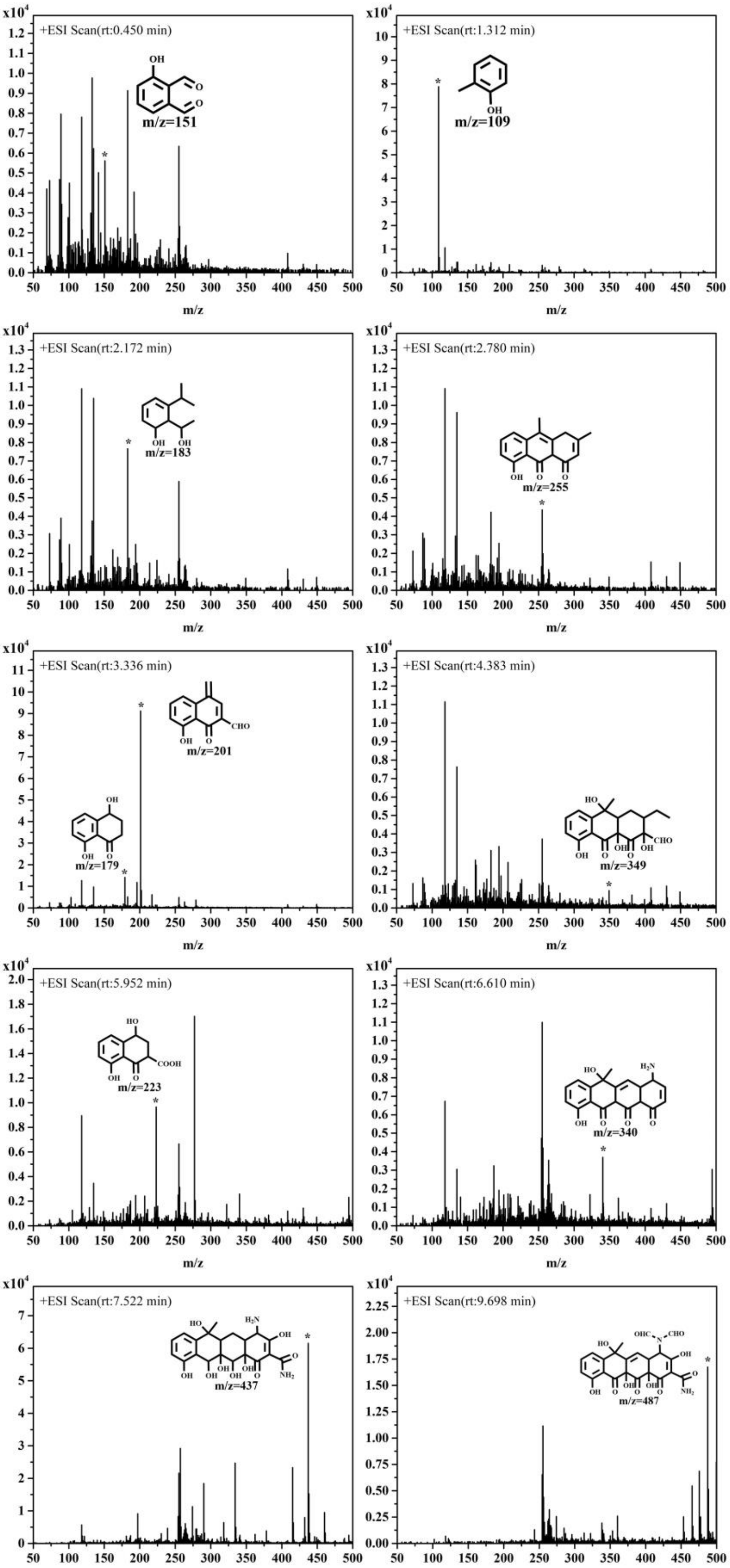

Figure S13. LC-MS spectra of the TCH intermediates.

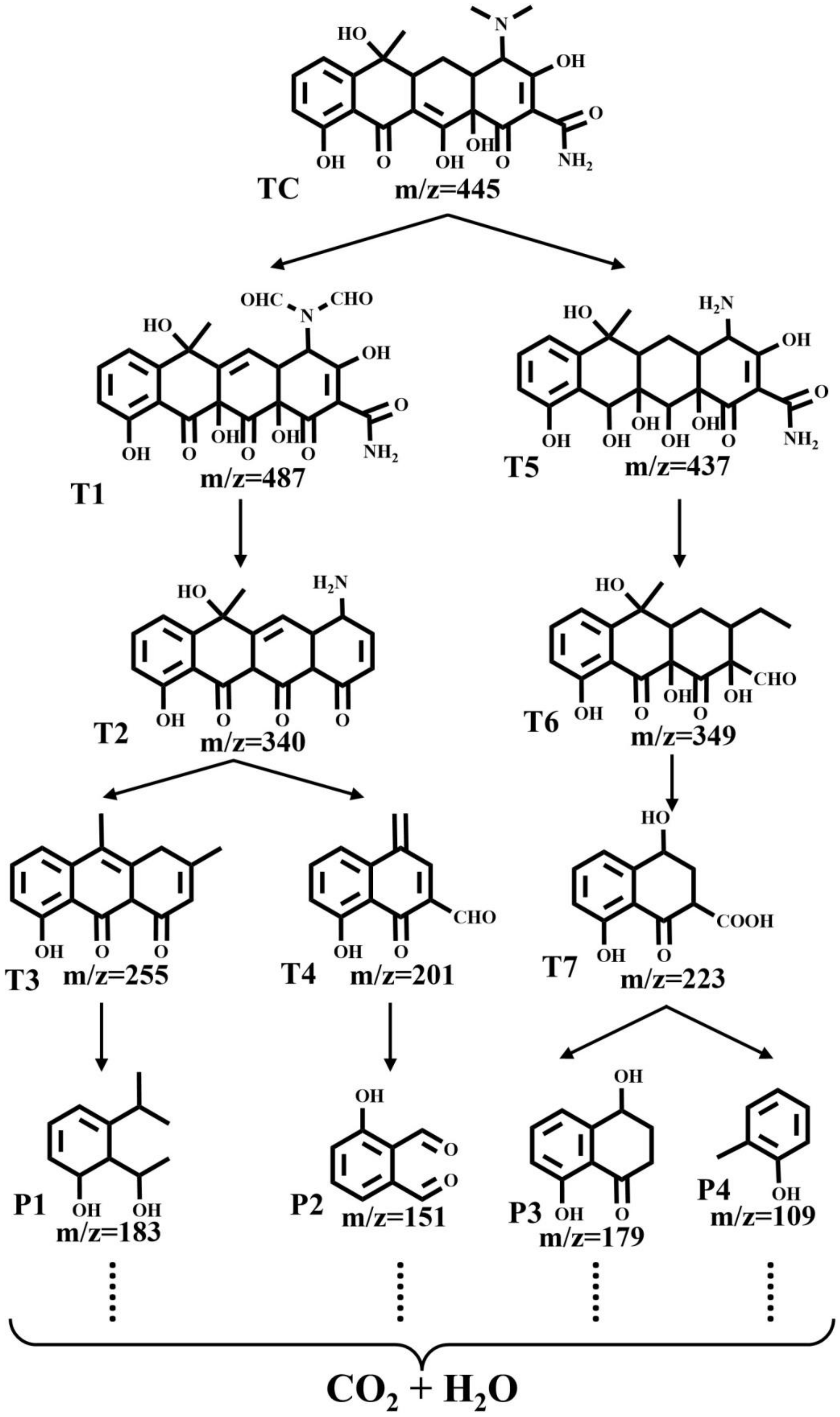

Figure S14. The proposed transformation pathways of TCH degradation.

References

(1) Bai, C.; Yang, G.; Zhang, S.; Deng, S.; Zhang, Y.; Chen, C.; He, J.; Xu, M.; Long, L. A synergistic system of electrocatalytic-anode/α-MnO_2/peroxymonosulfate for removing combined pollution of tetracycline and Cr(VI). *Chem. Eng. J.* **2021,** *423*, 130284.

(2) Luo, X.; Zheng, Z.; Greaves, J.; Cooper, W. J.; Song, W. Trimethoprim: kinetic and mechanistic considerations in photochemical environmental fate and AOP treatment. *Water Res.* **2012,** *46* (4), 1327-1336.
